# Anti-leukemia activity of a Hsp70 inhibitor and its hybrid molecules

**DOI:** 10.1038/s41598-017-03814-6

**Published:** 2017-06-14

**Authors:** Seong-Hyun Park, Won-Je Kim, Hui Li, Wonil Seo, Sang-Hyun Park, Hwan Kim, Sang Chul Shin, Erik R. P. Zuiderweg, Eunice EunKyeong Kim, Taebo Sim, Nak-Kyoon Kim, Injae Shin

**Affiliations:** 1 National Creative Research Initiative Center for Biofunctional Molecules, Department of Chemistry, Yonsei University, Seoul, 03722 Korea; 2 Advanced Analysis Center, Korea Institute of Science and Technology (KIST), Seoul, 02792 Korea; 3 Chemical Kinomics Research Center, Korea Institute of Science and Technology (KIST), Seoul, 02792 Korea; 4 Biomedical Research Institute, Korea Institute of Science and Technology (KIST), Seoul, 02792 Korea; 5 Department of Biological Chemistry, The University of Michigan, Ann Arbor, MI 48109 USA; 6 KU-KIST Graduate School of Converging Science and Technology, Korea University, Seoul, 02841 Korea

## Abstract

In this study we examined the anti-leukemia activity of a small molecule inhibitor of Hsp70 proteins, apoptozole (Az), and hybrids in which it is linked to an inhibitor of either Hsp90 (geldanamycin) or Abl kinase (imatinib). The results of NMR studies revealed that Az associates with an ATPase domain of Hsc70 and thus blocks ATP binding to the protein. Observations made in the cell study indicated that Az treatment promotes leukemia cell death by activating caspase-dependent apoptosis without affecting the caspase-independent apoptotic pathway. Importantly, the hybrids composed of Az and geldanamycin, which have high inhibitory activities towards both Hsp70 and Hsp90, exhibit enhanced anti-leukemia activity relative to the individual inhibitors. However, the Az and imatinib hybrids have weak inhibitory activities towards Hsp70 and Abl, and display lower cytotoxicity against leukemia cells compared to those of the individual constituents. The results of a mechanistic study showed that the active hybrid molecules promote leukemia cell death through a caspase-dependent apoptotic pathway. Taken together, the findings suggest that Hsp70 inhibitors as well as their hybrids can serve as potential anti-leukemia agents.

## Introduction

**Figure 1:**
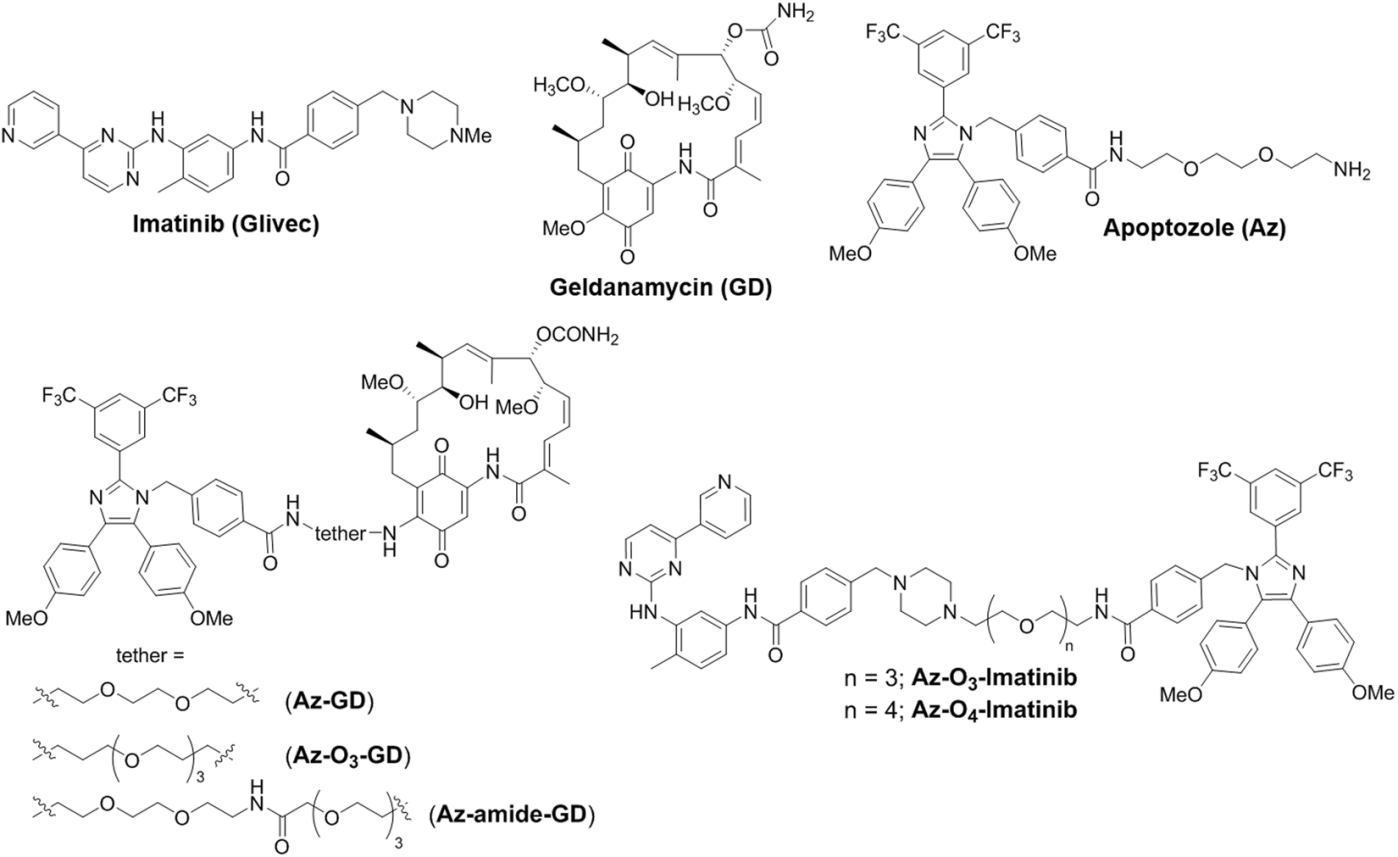
Chemical structures of substances used in this study.

Leukemia is a class of cancers, which cause the increased number of abnormal white blood cells. Imatinib (or Glivec), a selective Abl kinase inhibitor (Fig. 1), is a highly efficacious drug to treat early-phase chronic myeloid leukemia which expresses an oncogenic Bcr-Abl fusion protein with a constitutively active Abl kinase^1, 2^. However, late-phase chronic myeloid leukemia becomes resistant to imatinib by expressing various Abl mutants^3^. The heterogeneity of leukemia caused by gene mutations and the status of patients with regards to the stage of leukemia reduce the efficacy of imatinib. Therefore, novel anti-leukemia agents that display broad selectivity towards a wide range of patients are urgently needed^4^.

Members of the Hsp70 proteins exhibit ATP-dependent chaperone activities, including protein folding, degradation of misfolded proteins, blocking denatured protein aggregation, and protein translocation^5, 6^. The two major members of cytosolic Hsp70 proteins are constitutive Hsc70 and inducible Hsp70. It is known that inducible Hsp70 is greatly produced in both solid and hematological tumors, a phenomenon that leads to an enhancement in cancer cell survival^7, 8^. The increased level of Hsp70 expression also correlates with resistance of cancers to chemotherapeutic agents, including imatinib^9, 10^. Moreover, simultaneous attenuation of the expression of both inducible Hsp70 and constitutive Hsc70 promotes apoptotic death of cancer cells without affecting normal cells^11^. As a consequence, Hsp70 proteins are potent targets for cancer diagnosis and prognosis, and their inhibitors are potential chemotherapeutic agents for treatment of various cancers^12, 13^.

Another class of ATP-dependent molecular chaperone, Hsp90, is known to bind and stabilize various cancer-associated client proteins, including Bcr-Abl^14, 15^. Thus, targeting Hsp90 with small molecule inhibitors represents yet another promising approach to the treatment of tumors^16, 17^. For example, geldanamycin (Fig. 1), which associates with the ATP binding site of Hsp90 and blocks its activity, is a candidate for anticancer therapy^18, 19^. However, the results of previous investigations indicate that inhibition of Hsp90 in itself is insufficient to bring about cancer cell death because exposure to geldanamycin or its synthetic derivatives induces upregulation of Hsp70^20^. This upregulation leads to a decrease in the anticancer activities of Hsp90 inhibitors. Therefore, dual inhibition of Hsp90 and Hsp70 proteins should be an important therapeutic strategy to generate efficacious anticancer agents^21, 22^.

Recently we showed that apoptozole (Az, Fig. 1), a small molecule inhibitor of both Hsc70 and Hsp70^23–27^, induces death of various solid tumor cells^25^. Guided by this finding, we designed an investigation to evaluate the anti-leukemia activity of Az. In addition, based on observations that unimolecular dual inhibitors with dual activities often have enhanced therapeutic efficacies relative to the individual components^28, 29^, we designed hybrids of Az. Specifically, hybrids in which Az is covalently linked to an inhibitor of either Hsp90 (geldanamycin) or Abl (imatinib) were synthesized and evaluated for their leukemia cell death activities. The results of our effort demonstrate that Az induces leukemic cell death and that its hybrids containing geldanamycin exhibit an improved anti-leukemia efficacy compared to that of Az or geldanamycin.

## Results

### NMR studies

**Figure 2:**
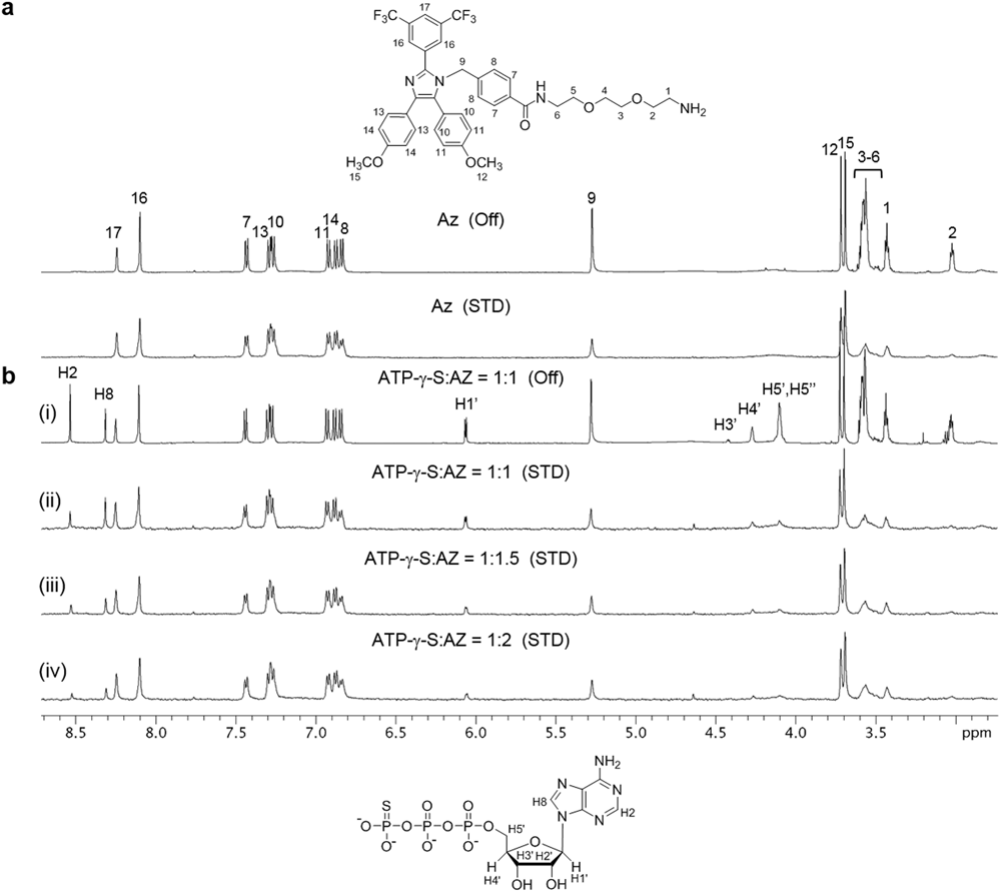
1D proton STD NMR spectra. (**a**) Off-resonance (top) and STD spectra (bottom) of a mixture of an ATPase domain of Hsc70 with Az. Numbers indicate the position of the protons in Az. (**b**) Off-resonance (i) and STD spectra (ii–iv) of a solution of an ATPase domain of Hsc70 mixed with ATP-γ-S and Az. The H2′ resonance of the ATP-γ-S is not observed due to the overalp with residual water signal (4.7 ppm) in the sample.

We previously showed that Az inhibits the ATPase activities of both Hsc70 and Hsp70, with high amino acid sequence and structural similarities, by binding to their ATPase domains^24, 25^. However, the detailed mode of Az binding to the proteins has not been elucidated. To gain information about the molecular basis of Az binding to Hsp70 proteins, NMR studies were carried out on a complex of Az with an ATPase domain (1-386 residues) of human Hsc70. Saturation transfer difference (STD) NMR studies were conducted initially to obtain information on Az binding to the ATPase domain of Hsc70^30^. The aromatic protons of Az show large STD signals (Fig. 2a)^24, 25^, suggesting that the aromatic rings in Az are responsible for major interactions with Hsc70. Next, we evaluated whether Az affects binding of ATP to the ATPase domain. For STD experiments, less hydrolytic ATP-γ-S was used in place of ATP. The results of NMR studies with a mixture of Hsc70, Az and ATP-γ-S showed that the STD NMR signals of ATP-γ-S are gradually attenuated as Az concentrations increase (Fig. 2b). This finding provides evidence that Az blocks ATP binding to the ATPase domain of Hsc70.

To identify the potential Az-binding site to the ATPase domain of Hsc70, chemical shift perturbation (CSP) experiments were carried out. In a CSP NMR study, changes in chemical shifts of the backbone amide N-H in the protein that take place upon addition of a binding ligand are monitored. CSP is sensitive to structural changes and, as a result, residues containing protons that experience the largest chemical shift changes are generally considered to be residing in the ligand-binding site^31^. Because high concentrations of the protein and the ligand sometimes lead to undesired protein aggregation or precipitation, dynamic light scattering (DLS) analysis was first conducted to determine optimal concentrations of Az and the ATPase domain at which protein aggregation does not occur. The results showed that aggregates do not form in the mixture of 150 μM Az and 75 μM ATPase domain of Hsc70 (Supplementary Fig. S1), used for the CSP NMR study.

**Figure 3:**
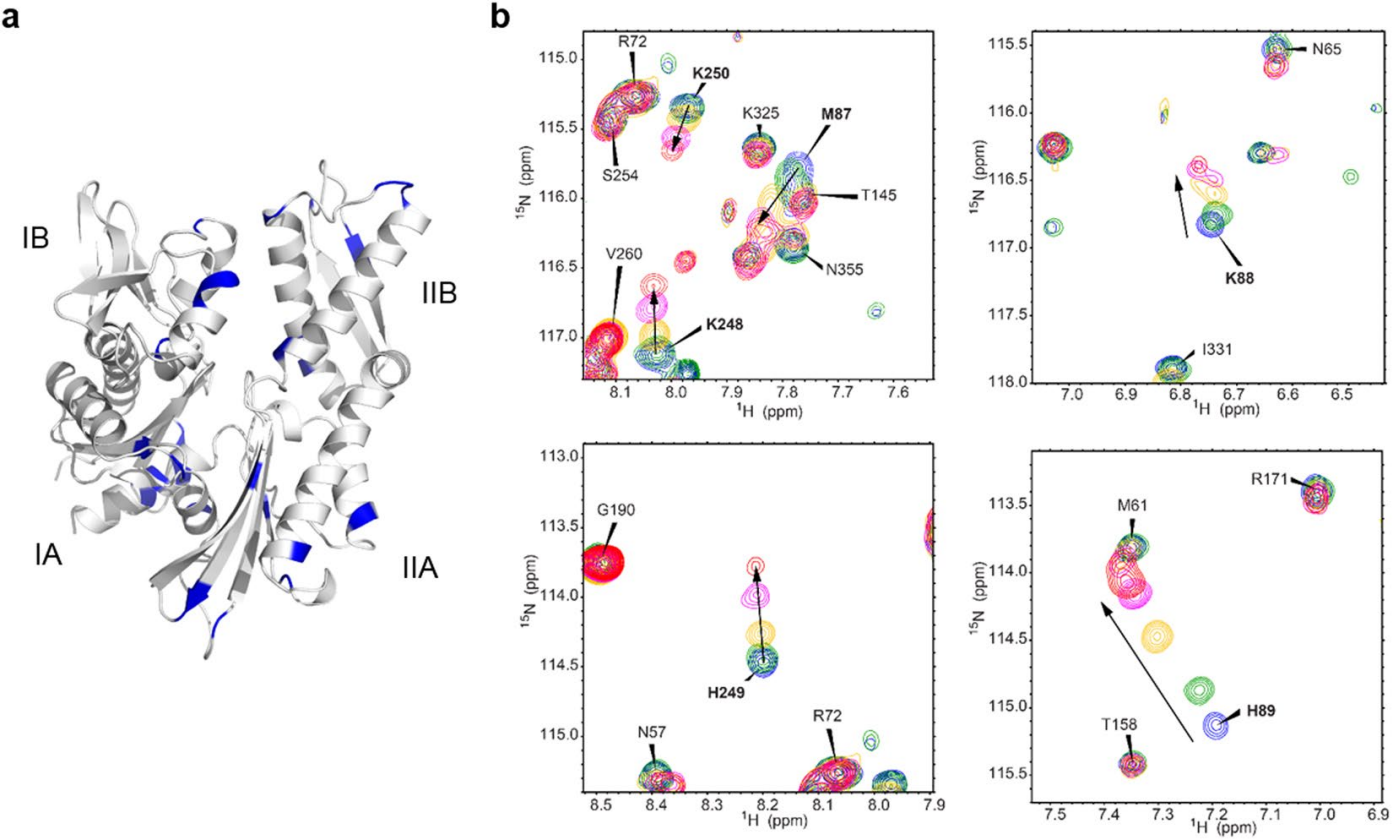
Chemical shift perturbation. (**a**) Residues with large chemical shift changes (Δδ_NH_ > 0.04 ppm) upon Az binding to an ATPase domain of Hsc70 were mapped in blue on the structure (PDB entry 2E8A). Subdomains IA, IIA, IB and IIB are shown in the structure (**b**) Chemical shift changes of M87-H89 and K248-K250 after addition of Az to an ATPase domain of Hsc70 are shown in 2D ^15^N-^1^H TROSY HSQC spectra. The ratios of protein to Az are 1:0 (blue), 1:0.5 (green), 1:1 (yellow), 1:1.25 (magenta) and 1:1.5 (red).

ADP-induced CSP was initially evaluated in ^15^N-^1^H TROSY HSQC spectra of a mixture of ^15^N-labeled ATPase domain and ADP. Large chemical shift changes were detected to take place for the amino acid residues in the ATP binding site and for many other residues owing to ADP-binding induced protein conformational changes (Supplementary Fig. S2), phenomena which are consistent with those seen in analysis of DnaK from *Thermus thermophiles* ^32^. Next, we investigated whether Az binds to the ATPase domain of Hsc70 by monitoring chemical shift changes of resonances associated with protons in Hsc70 that are caused by Az binding. Analysis of the NMR data revealed that the locations of the largely perturbed proton resonance (Δδ_NH_ > 0.04 ppm) following addition of Az were found to be comparable to those promoted by ADP (Fig. 3a and Supplementary Fig. S2a). Notably, several residues of Az-treated Hsc70, such as M87-H89 in domain IB and K248-K250 in domain IIB, exhibit large CSPs (Fig. 3b and Supplementary Fig. S2c), indicating that Az binds to this site. These findings indicate that Az has a Hsc70 binding site that is similar to that of ADP and, thus, that is positioned near the ATP binding site to block binding of ATP to the protein.

### Az induces apoptosis in leukemia cells

To assess anti-leukemia activity of Az, several human leukemia cells (HL-60 cells, acute myeloid leukemia; Jurkat cells, acute T-lymphocytic leukemia; KG-1 cells, acute myeloid leukemia; U937 cells, acute myeloid leukemia; THP-1 cells, acute monocytic leukemia; K562 cells, chronic myeloid leukemia) were incubated with Az for 48 h. As controls, leukemia cells were also incubated with the known anti-leukemia agents, geldanamycin and imatinib^19, 33^. An MTT assay was then performed to measure cell viabilities. The results revealed that Az has cytotoxic activities toward all the tested leukemia with IC_50_ values of 2–5 μM (Table 1 and Supplementary Fig. S3). In addition, Az has similar cell death activities against both acute and chronic leukemia cells. Moreover, the anti-leukemia activity of Az is similar to that of geldanamycin. Interestingly, Az displays a higher anti-leukemia activity against acute leukemia cells but a lower chronic leukemia cell death activity as compared to that of imatinib.

**Table 1:** IC_50_ values (μM) of compounds for leukemia cells (mean ± s.d., n = 3).

Cells	Az	GD	Imatinib	Az-GA	Az-O_3_-GD	Az-amide-GD	Az-O_3_-Imatinib	Az-O_4_-Imatinib
HL-60	2.80 ± 0.87	2.30 ± 0.74	9.60 ± 1.52	0.73 ± 0.22	0.74 ± 0.27	>10	>10	>10
KG-1	3.30 ± 0.71	3.20 ± 1.21	8.58 ± 1.61	0.91 ± 0.41	1.31 ± 0.30	>10	>10	>10
THP-1	3.70 ± 0.73	0.90 ± 0.33	8.41 ± 1.55	0.36 ± 0.12	0.62 ± 0.27	>10	>10	>10
U937	4.70 ± 0.77	4.80 ± 1.22	8.58 ± 1.02	1.12 ± 0.32	1.12 ± 0.44	>10	>10	>10
Jurkat	3.10 ± 0.61	4.80 ± 1.06	9.72 ± 1.49	0.89 ± 0.25	0.57 ± 0.18	>10	>10	>10
K562	3.60 ± 0.52	3.70 ± 1.11	0.35 ± 0.10	0.63 ± 0.21	0.72 ± 0.26	>10	4.3 ± 0.91	4.5 ± 0.88

Next, the cytotoxic effects of Az on wild-type and mutant Bcr-Abl expressing cells were evaluated. In this study Ba/F3 cells, stably producing either wild-type or imatinib-resistant mutant Bcr-Abl (T315I), were separately treated with Az, along with imatinib as a control. The results of MTT assays revealed that Az promotes similar cell death towards both types of cells producing wild-type and mutant Bcr-Abl (Supplementary Fig. S4 and Supplementary Table S1). Importantly, Az has a much higher cytotoxicity towards cells expressing mutant Abl than does imatinib (IC_50_ values, 2.2 vs. 37.4 μM). The findings show that unlike imatinib, Az stimulates the death of leukemia cells irrespective of the expression of wild-type and mutant Abl kinases.

To determine whether Az promotes leukemia cell death via apoptosis, acute leukemia HL-60 and chronic leukemia K562 cells were independently incubated with Az. The treated cells were then exposed to a mixed solution of fluorescein-annexin V and propidium iodide (PI) and flow cytometry analysis was conducted. Because cells treated with Az exhibit positive annexin V binding and positive PI uptake (Supplementary Fig. S5a), Az promotes apoptosis^34^. The size of the treated cells was also assessed by using flow cytometry. It was found that Az treatment leads to cell shrinkage to a great degree (Supplementary Fig. S6). This finding also indicates that leukemia cells treated with Az undergo apoptosis. We also investigated if the mitochondrial membrane potential in cells treated with Az is disrupted. To test this possibility, HL-60 and K562 cells were exposed to Az and subsequently stained with a membrane potential sensitive JC-1 probe^35^. The intensity of red fluorescence derived from JC-1 in the treated cells was found to be significantly lower than that of untreated cells, indicating that Az causes a reduction of the mitochondrial membrane potential (Supplementary Fig. S5b). This serves as another evidence for apoptosis induced by Az. Furthermore, an increase in DNA fragments was found to take place in cells treated with Az (Supplementary Fig. S7). Collectively, the results clearly demonstrate that Az induces apoptotic cell death in both chronic and acute leukemia cells.

### Leukemia cell death stimulated by Az takes place through caspase-dependent apoptosis

A study was conducted to determine if apoptosis of leukemia cells promoted by Az takes place through a caspase-dependent and/or caspase-independent pathway. Caspase-dependent apoptosis includes the release of cytochrome c from mitochondria into the cytosol to form the apoptosome after association with Apaf-1 (Supplementary Fig. S8a)^36, 37^. Subsequently, caspase-9 is produced from procaspase-9 through proteolytic cleavage in the complex of cytochrome c and Apaf-1. The process leads to cleavage of procaspase-3 to form caspase-3. It is known that Hsp70 suppresses apoptosome formation by the direct association of Hsp70 with Apaf-1^38^.

**Figure 4:**
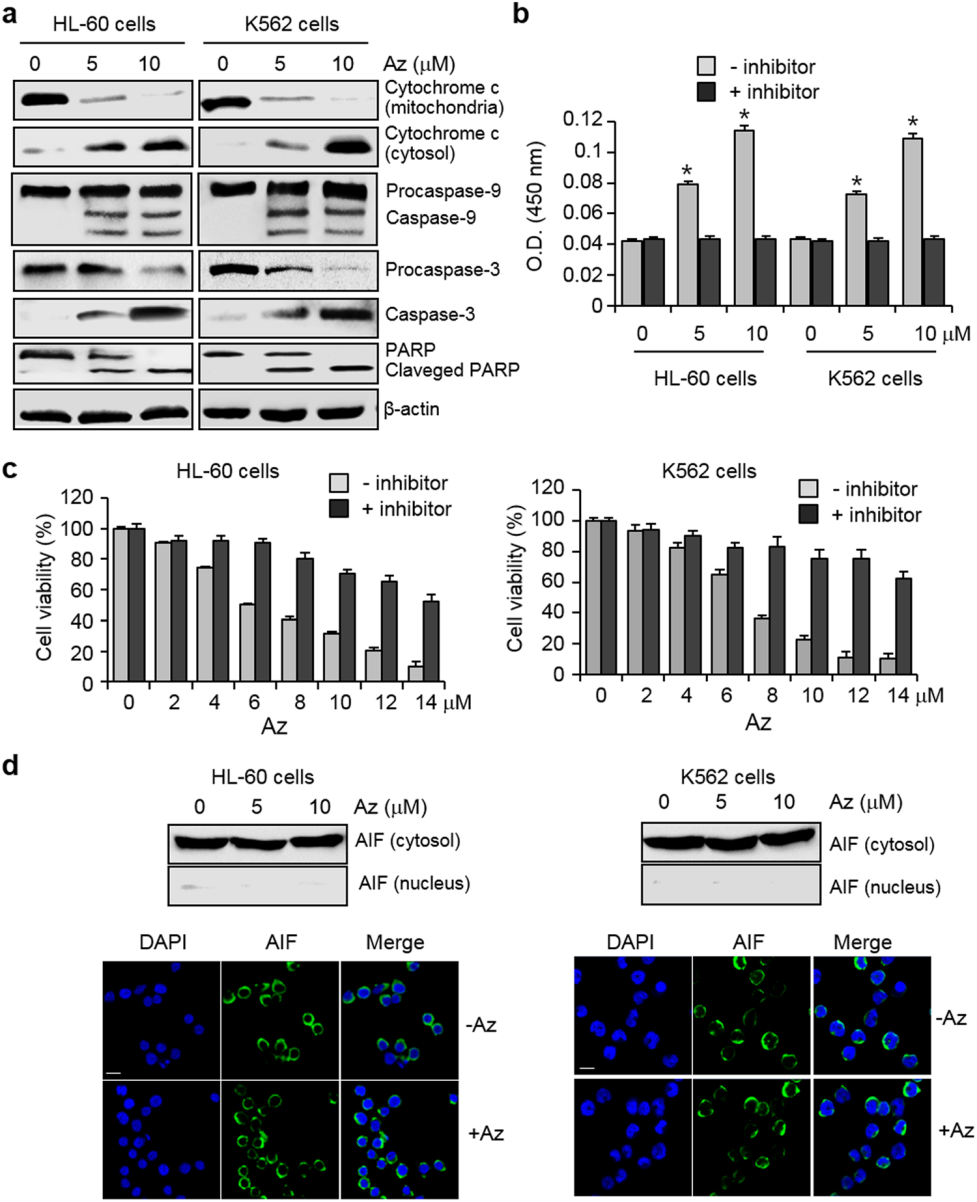
Az induces caspase activation but does not promote the AIF-associated, caspase-independent apoptosis. (**a**) HL-60 and K562 cells were treated with Az for 24 h and the indicated proteins were immunoblotted by using appropriate corresponding antibodies. β-Actin was used as a loading control. (**b**) Caspase activities of lysates of HL-60 and K562 cells treated with Az for 24 h were measured by using Ac-DEVD-pNA in the absence or presence of 20 μM Ac-DEVD-CHO (mean ± s.d., n = 3). *P < 0.05. (**c**) HL-60 and K562 cells were pre-incubated with 20 μM ZVAD-FMK for 3 h, and then treated with various concentrations of Az for 24 h. Effect of a caspase inhibitor on cell survival was determined by using an MTT assay (mean ± s.d., n = 3). (**d**) HL-60 and K562 cells were treated with Az for 24 h. (Top) Immunocytochemistry and (bottom) western blotting were performed by using an anti-AIF antibody. The nuclei were stained with DAPI (scale bar = 10 μm).

With this information in mind, we tested if Az enhances the cytochrome c release from mitochondria into the cytosol in leukemia cells. The levels of cytochrome c in cytosolic and mitochondrial fractions of HL-60 and K562 cells after treatment with Az were determined by using western blot analysis. Cytochrome c was found to be released from mitochondria into the cytosol after Az treatment (Fig. 4a). We next determined if the binding of Hsp70 to Apaf-1 is suppressed by Az during apoptosis. For this purpose, HL-60 and K562 cells were exposed to Az, and a complex of Hsp70 and Apaf-1 was then immunoprecipitated with Hsp70 antibody. It was revealed that Az inhibits the interaction of Hsp70 with Apaf-1 but it does not influence the translational levels of Hsp70 and Apaf-1 (Supplementary Fig. S9). This finding indicates that Az suppresses the association of Hsp70 with Apaf-1 for which the ATPase activity of Hsp70 is critical^38^.

Because Az inhibits the association of Hsp70 with Apaf-1, caspase associated proteolytic activities should increase in cells treated with Az. To determine whether this event occurs, caspase activities of HL-60 and K562 cells treated with Az were measured by using the colorimetric substrate, Ac-DEVD-pNA (pNA, p-nitroaniline). The caspase activity was found to increase in the Az treated cells (Fig. 4b). In contrast, caspase activity was greatly reduced in the presence of Ac-DEAD-CHO (a caspase inhibitor). We also examined whether leukemia cells are protected from the effect of Az by ZVAD-FMK (benzyloxycarbonyl-Val-Ala-Asp(OMe) fluoromethyl ketone, a cell-permeable inhibitor of pan-caspases). HL-60 and K562 cells were pre-incubated with ZVAD-FMK for 3 h and subsequently exposed to Az. The results of an MTT assay showed that ZVAD-FMK significantly protects cells against Az induced death (Fig. 4c).

Apaf-1 mediated, caspase-dependent apoptosis promoted by Az in leukemia cells should lead to activation of proteolysis of procaspase-9 to produce caspase-9^38^. To check this, western blot analysis was performed in the cells treated with Az. Cleaved caspase-9 was detected in the treated cells as well as caspase-3 was generated from procaspase-3 (Fig. 4a). Furthermore, the endogenous caspase substrate PARP is also cleaved in the treated cells (Fig. 4a). These results clearly indicate that Az enhances apoptosis of leukemia cells in a caspase-dependent manner.

Apoptosis-inducing factor (AIF) is known to be translocated from the cytosol to the nucleus during caspase-independent apoptosis (Supplementary Fig. S8b)^39^. In addition, it is also known that Hsp70 interacts with AIF directly and this interacting event results in blocking translocation of AIF to the nucleus and, in turn, prevents caspase-independent apoptosis^40^. Because of these phenomena, we tested if Az has an influence on AIF-mediated caspase-independent apoptosis. To probe this issue, HL-60 and K562 cells were exposed to Az and the levels of AIF in the cytosolic and nuclear fractions were then determined. AIF translocation to the nucleus was not observed in Az-treated cells, based on western blot analysis (Fig. 4d). Additional evidence to support this conclusion was obtained from immunocytochemical analysis of leukemia cells treated with Az (Fig. 4d). The observations show that Az does not affect the interaction of Hsp70 with AIF for which the ATPase activity is not required^41^. As a result, Az does not promote apoptosis of leukemia cells in an AIF-mediated, caspase-independent manner. Collectively, the results show that Az enhances leukemia cell death by activating caspases without affecting the AIF-associated apoptotic pathway.

### Design and synthesis of hybrid molecules consisting of Az and an inhibitor of either Hsp90 or Abl

Development of single target drugs is a predominant strategy used in drug discovery. However, a gradual shift has taken place toward a new approach in which hybrid drugs are designed to modulate multiple targets simultaneously in order to enhance therapeutic efficacy relative to single-target drugs. Thus, in the current effort we explored this approach using hybrids that are designed to target both Hsp70 and Hsp90 or both Hsp70 and Abl.

**Figure 5:**
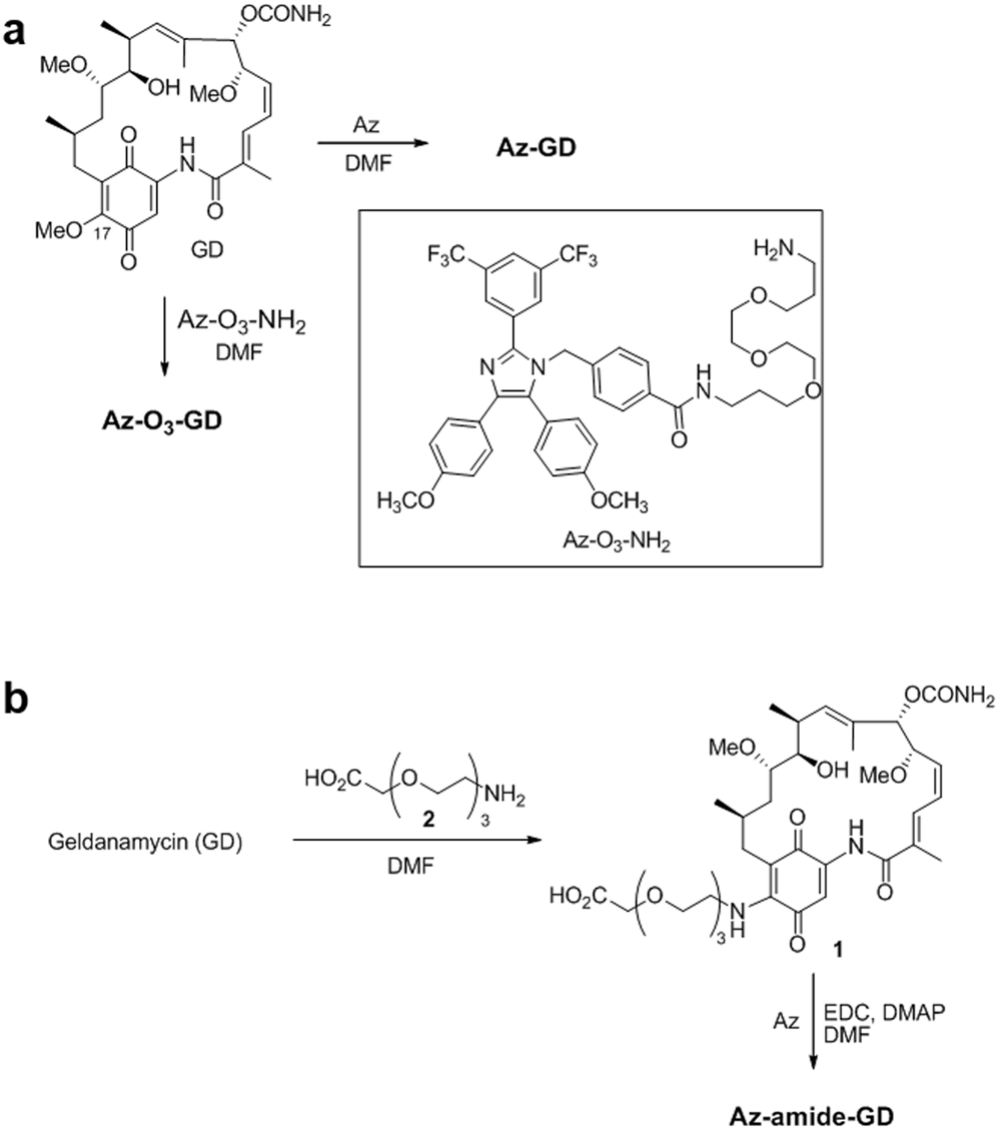
Routes for synthesis of hybrid molecules consisting of Az and geldanamycin (GD) to give (**a**) Az-GD and Az-O_3_-GD and (**b**) Az-amide-GD.

**Figure 6:**
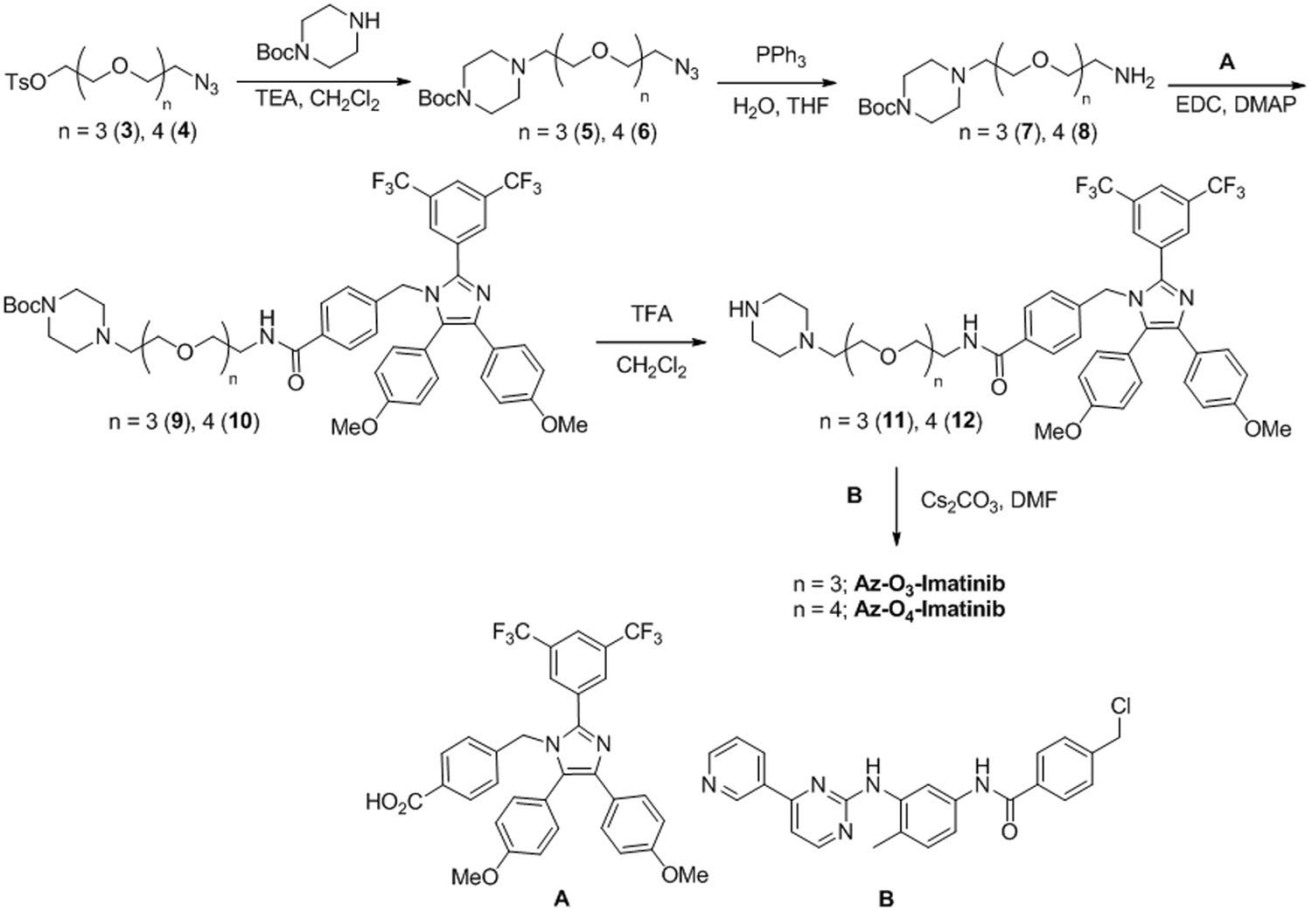
Routes for synthesis of hybrid molecules consisting of Az and imatinib to yield Az-O_3_-Imatinib and Az-O_4_-Imatinib.

Hybrids, in which Az is covalently linked to geldanamycin (GD) and imatinib, were synthesized utilizing the routes displayed in Fig. 5 and 6. In routes to prepare Az-geldanamycin hybrids (Fig. 5), Az and its triethylene glycol analog Az-O_3_-NH_2_ reacted with GD at the C-17 position to produce the respective covalent adducts Az-GD and Az-O_3_-GD (Fig. 5a)^42^. In addition, the GD derivative **1**, generated by reaction of GD with linker **2**, was coupled to Az under amide coupling conditions to yield Az-amide-GD (Fig. 5a).

To prepare the Az-imatinib hybrids, Az-O_3_-Imatinib and Az-O_4_-Imatinib (Fig. 1), the carboxylic acid containing imidazole derivative **A** (Fig. 6) was coupled to **7** and **8** to afford the respective **9** and **10**. Removal of the Boc-groups from **9** and **10** produced the corresponding secondary amines **11** and **12**, which underwent nucleophilic substitution reactions with the benzyl chloride containing imatinib derivative **B** to give Az-O_3_-Imatinib and Az-O_4_-Imatinib, respectively. Selection of these Az-imatinib targets was based on the crystal structure of a complex of Abl with imatinib, which showed that *N*-methylpiperazine moiety of imatinib is located on the outside of Abl^43^. The synthesized hybrid molecules were characterized by using ^1^H/^13^C NMR and MS techniques.

### Anti-leukemia activity of hybrid molecules

Inhibition by the hybrids on the ATPase activities of Hsp70 and Hsp90 was determined by using a PiColorLock assay and on the kinase activity of Abl by using a radiometric assay. The results of these assays indicate that both Az-GD and Az-O_3_-GD suppress the ATPase activity of Hsp70 to a similar degree that Az (Supplementary Fig. S10a). In contrast, Az-amide-GD has a lower inhibitory activity towards Hsp70 than Az. It was also found that Az-O_3_-Imatinib and Az-O_4_-Imatinib are much weaker inhibitors of Hsp70 compared to Az itself. The findings suggest that the nature of modifications has a profound effect on the inhibitory activities of the Az-hybrids towards Hsp70.

Three Az-GD hybrids, Az-GD, Az-O_3_-GD and Az-amide-GD, were shown to abrogate the ATPase activity of Hsp90 to the similar degree that GD itself does, indicating that modification of GD has little influence on its inhibitory activity (Supplementary Fig. S10b). In contrast, the imatinib containing hybrids, Az-O_3_-Imatinib and Az-O_4_-Imatinib, have significantly reduced inhibitory activities towards Abl kinase compared to imatinib itself (Supplementary Fig. S10c). This finding suggests that attachment of a relatively large group to the piperazine moiety of imatinib has an adverse influence on inhibition of Abl kinase.

Next, to evaluate leukemia cell death activities of the hybrids, several leukemia cells (HL-60, KG-1, THP-1, U937, Jurkat and K562 cells) were exposed to the hybrids, along with individual inhibitors (Az, GD and imatinib) as controls. The results of an MTT assay showed that Az-amide-GD, Az-O_3_-Imatinib and Az-O_4_-Imatinib, which have low inhibitory activities towards Hsp70 and Abl, display poorer cytotoxicity towards all of the tested leukemia cells as compared to those of Az, GD and imatinib (Table 1 and Supplementary Fig. S11). Importantly, Az-GD and Az-O_3_-GD, which have high inhibitory activities towards both Hsp70 and Hsp90, exhibit greatly enhanced cytotoxicities against all the leukemia cells relative to those of Az and GD. The results indicate that hybrids consisting of Hsp70 and Hsp90 inhibitors linked through a proper tether have enhanced anti-leukemia activities relative to the individual inhibitors.

### Az-O_3_-GD stimulates leukemia cell death via caspase-dependent apoptosis

In the final phase of this investigation, we determined whether leukemia cell death induced by the Az-GD hybrids takes place via a caspase-dependent and/or caspase-independent apoptotic pathway. Because Az-GD and Az-O_3_-GD have similar anti-leukemia activities, the latter was employed in this study. HL-60 and K562 cells were incubated for 24 h with Az-O_3_-GD, along with Az and GD as controls, and then incubated with a mixed solution of annexin V and PI as well as JC-1. Observations of positive annexin V and positive PI uptake, cell shrinkage and a significant decrease in the intensity of red fluorescence from JC-1 using flow cytometry analysis indicate that, just like Az and GD, Az-O_3_-GD induces apoptosis of the cells (Supplementary Fig. S12).

**Figure 7:**
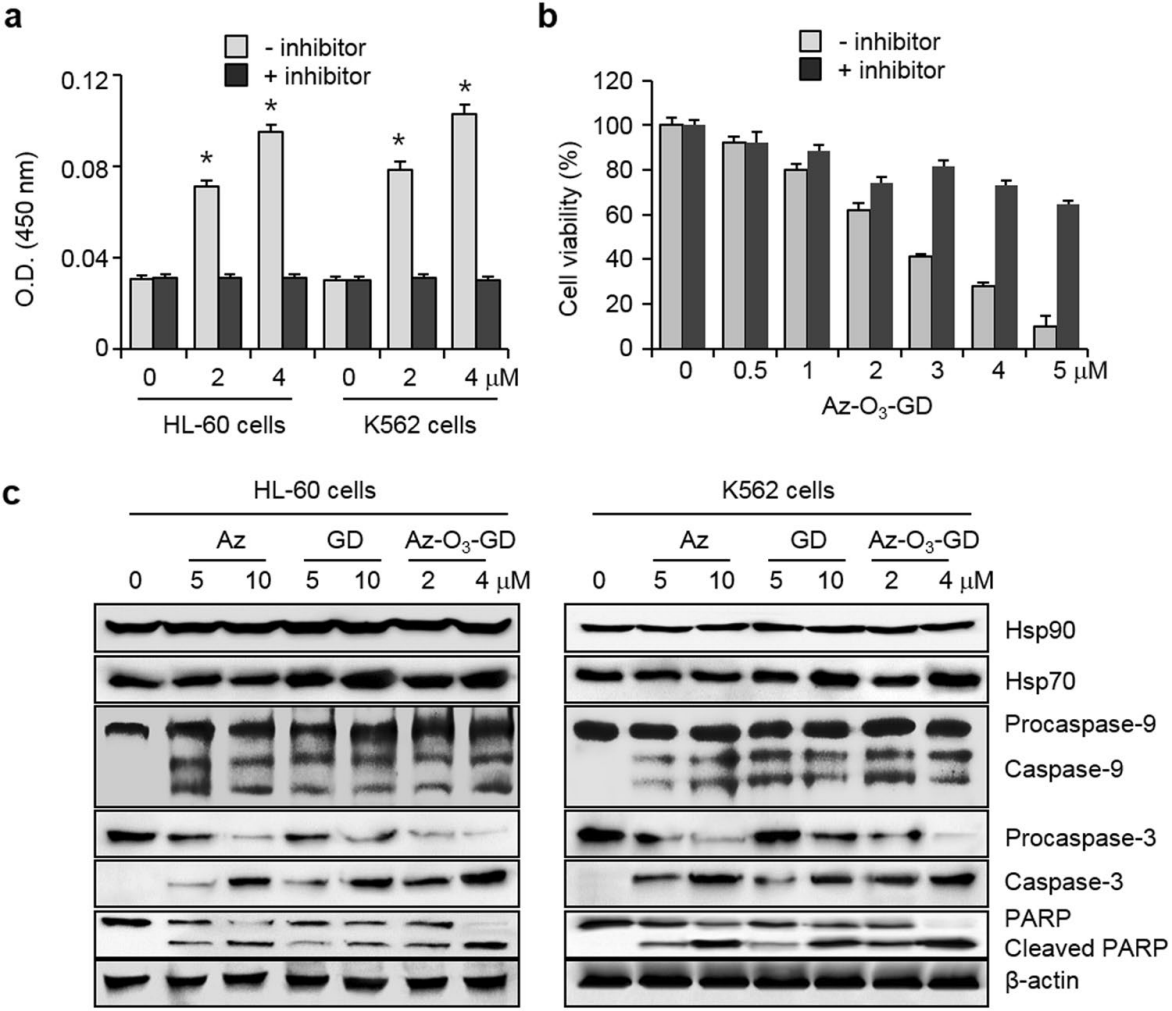
Az-O_3_-GD induces caspase-dependent apoptosis in leukemia cells. (**a**) Caspase activities of lysates of HL-60 and K562 cells treated with Az-O_3_-GD for 24 h were measured by using acetyl-DEVD-pNA in the absence or presence of 20 μM Ac-DEVD-CHO (mean ± s.d., n = 3). *P < 0.05. (**b**) HL-60 cells were pre-incubated with 20 μM ZVAD-FMK for 3 h, and then treated with various concentrations of Az-O_3_-GD for 24 h. Effect of a caspase inhibitor on cell survival was determined by using an MTT assay (mean ± s.d., n = 3). (**c**) Upper panels: HL-60 and K562 cells were treated with each compound for 24 h and the indicated proteins were immunoblotted by using appropriate corresponding antibodies.

Given that Az-O_3_-GD treatment induces apoptosis, caspase activities of lysates of HL-60 and K562 cells exposed to Az-O_3_-GD were determined by employing Ac-DEVD-pNA. Caspase activities were found to increase in the treated cells but to greatly decrease in the presence of Ac-DEAD-CHO (Fig. 7a). We also examined whether leukemia cells are protected against the effect of Az-O_3_-GD by ZVAD-FMK. To this end, HL-60 and K562 cells pre-treated with ZVAD-FMK for 3 h were exposed to Az-O_3_-GD. The results of an MTT assay revealed that leukemia cell death induced by Az-O_3_-GD is greatly attenuated in the presence of the pan-caspase inhibitor (Fig. 7b and Supplementary Fig. S13).

Because Az-O_3_-GD promotes caspase activation in leukemia cells, it was anticipated that procaspase-9, procaspase-3 and PARP would be cleaved in treated cells. To test this possibility, HL-60 and K562 cells were exposed to Az-O_3_-GD, along with Az and GD as controls and western blot analysis was then carried out. It was revealed that caspase-9 and caspase-3 are generated from procaspase-9 and procaspase-3, respectively, in the treated cells, and that cleaved PARP is also seen (Fig. 7c). Unlike Az which does not affect the translational levels of Hsp70 and Hsp90, Az-O_3_-GD treatment leads to upregulation of Hsp70, a phenomenon which is seen in cells exposed to GD alone^44^. Although Az-O_3_-GD upregulates Hsp70, the presence of the Hsp70 inhibitor in this hybrid causes its cytotoxicity against leukemia cells to be higher than that of GD. Finally, we examined the effect of Az-O_3_-GD on AIF-mediated caspase-independent apoptosis. To this end, HL-60 and K562 cells were incubated with Az-O_3_-GD for 24 h. The results of immunostaining of the treated cells revealed that Az-O_3_-GD does not elicit the translocation of AIF to the nucleus (Supplementary Fig. S14). Collectively, the observations demonstrate that Az-O_3_-GD promotes caspase-dependent apoptosis of leukemia cell, a phenomenon which is observed in cancer cell death induced by Az and GD^25, 45^.

## Discussion

Much progress has been made in the discovery of efficacious chemotherapeutic agents for cancer treatment and these advances have led to substantially increased survival rates for patients. In particular, imatinib, a selective inhibitor of Abl kinase, has greatly contributed to the treatment of leukemia. However, resistance of imatinib by mutation of the Abl gene stimulated us to develop novel efficacious anti-leukemia agents that have broad ranges of selectivity. In the investigation described above, we demonstrated that Az promotes leukemia cell death by activating caspase-dependent apoptosis without affecting AIF-mediated caspase-independent apoptosis. Importantly, we showed that hybrid molecules comprised of Az and geldanamycin linked through oligoethylene glycol have enhanced anti-leukemia activities compared to those of the individual substances. The findings indicate that Az will continue to play a special role in blood cancer research and that the efficacy of Az in the treatment of cancers can be augmented by utilizing a combination of Az with other anticancer agents to simultaneously target two causal mechanisms of cancers. Finally, it is anticipated that the strategy involving the design of hybrid molecules to target multiple proteins that are involved in cancers will be expanded as a therapeutic approach to treat cancers.

## Materials and Methods

### Chemicals

Geldanamycin (GD) was purchased from Hubei Widely Chemical Technology Co., Ltd. (China) and Imatinib from LC laboratories (USA). Hybrid molecules, Az-GD, Az-O_3_-GD, Az-amide-GD, Az-O_3_-imatinib and Az-O_4_-imatinib, were prepared and their analytical data were shown in Supporting Information.

### Saturation transfer difference (STD) NMR experiments

For STD experiments, 10 μM of an ATPase domain of Hsc70, 1 mM of ATP-γ-S and Az (a phosphate salt form) were used. For competition studies between ATP-γ-S and Az, STD of the sample with 5 μM ATPase domain of Hsc70 and 500 μM ATP-γ-S was initially measured, and further titrated with Az up to 1 mM. The ratios of ATP-γ-S to Az were 1:0, 1:1, 1:1.5, and 1:2. All STD NMR spectra were obtained using 20 mM solutions of deuterated Tris buffer with 5% (v/v) DMSO-d_6_ (pD 7.2). The STD signals were recorded with 2 sec transfer delay and 1,024 scans using a 600 MHz spectrometer (Agilent DD2) at 298 K (on-resonance irradiation, −1.0 ppm; off-resonance irradiation, 31.6 ppm).

### Chemical shift perturbation NMR study

Resonance assignments for backbone amide nitrogens and protons of a ligand-free ATPase domain of Hsc70 were accomplished by back-tracing the chemical shift values from NMR data of an ADP-bound ATPase domain. First, previously determined chemical shift values of ADP-bound Hsc70 (10 mM ADP state) were used as a starting point of the resonance assignments^46^. Upon reducing the final concentrations of ADP (10 mM, 200 μM, 50 μM, and 0 M), the N-H chemical shift changes of ^15^N-labeled Hsc70 (100 μM) in an NMR buffer (20 mM Tris-HCl, 25 mM KCl, 5 mM MgCl_2_, 5 mM K_3_PO_4_, 10% (v/v) D_2_O, pH 7.2) were monitored. For Az titrations to Hsc70 in the ligand-free state, ^15^N-labeled Hsc70 (75 μM) in the NMR buffer with 1% DMSO-d_6_ was used. The 1% DMSO was used to increase the solubility of Az in protein solution, but the effect of addition of DMSO on the chemical shift perturbation was negligible. The ratios of Hsc70 (the ligand-free state) to Az (a phosphate salt form) were 1:0, 1:0.5, 1:1, 1:1.25, and 1:1.5. The chemical shift changes during the titrations were monitored in 2D TROSY (Transverse Relaxation Optimized SpectroscopY) ^15^N-^1^H HSQC spectra of Hsc70. The chemical shift difference (Δδ_NH_) was calculated by using the equation of Δδ_NH_ = [(Δδ_H_^2^ + (Δδ_N_/5)^2^)/2]^1/2^. All spectra were measured on an 800 MHz NMR spectrometer (Bruker) with a cryogenic probe. The NMR data were processed with Topspin 3.5 pl2 (Bruker) and analyzed with Sparky 3.114.

### Cell culture

HL-60, KG-1, Jurkat, U937, THP-1 and K562 cells were cultured in RPMI 1640 (Invitrogen) supplemented with 10% FBS, penicillin (50 units/mL) and streptomycin (50 units/mL). Ba/F3 cells (murine interleukin-3 dependent pro-B cells) producing wild-type and mutant Bcr-Abl (T315I) were cultured in RPMI 1640 supplemented with 10% FBS, penicillin (50 units/mL), streptomycin (50 units/mL) and interleukin-3 (10 ng/mL).

### Determination of cell death

Cells were exposed to each compound (a HCl salt form) at various concentrations for indicated time periods (24 or 48 h) in culture media. MTT assays were conducted according to known procedures. The absorbance at 570 nm was determined by employing a microplate reader (TECAN, Infinite® 200 PRO).

### Flow cytometry

HL-60 and K562 cells were incubated with each compound for 24 h in culture media. Cells were re-suspended with 500 μL binding buffer (10 mM HEPES, 1.4 M NaCl and 2.5 mM CaCl_2_, pH 7.5) and then incubated with the FITC-annexin V apoptosis detection kit with PI according to the protocol provided by the manufacturer. Flow cytometry was carried out by using a BD FACSVerse™ instrument (BD Biosciences) and the data were analyzed by using a FlowJo™ software (BD Biosciences).

Cell size measurement: HL-60 and K562 cells were treated with each compound for 24 h. The cell size was analyzed with a flow cytometer (excitation: a 488-nm argon laser) by measuring distribution on a forward scatter *versus* side scatter dot plot.

JC-1 staining: HL-60 and K562 cells were treated with each compound and then with 2.5 μg/mL JC-1 (Anaspec) in PBS for 15 min. Flow cytometry was conducted with a BD FACSVerse™ instrument (the red fluorescence signal: λ_ex_ = 550 nm, λ_em_ = 600 nm; the green fluorescence signal: λ_ex_ = 485 nm, λ_em_ = 535 nm) and the data were analyzed using a FlowJo™ software.

### Caspase activity assay

HL-60 and K562 cells treated with each compound for 24 h were lysed in a buffer containing 50 mM HEPES (pH 7.4), 5 mM CHAPS and 5 mM DTT. Assay buffer containing 20 mM HEPES (pH 7.4), 0.01% CHAPS, 5 mM DTT and 2 mM EDTA was added to cell lysates. Caspase activity was measured in the absence or presence of acetyl-DEVD-pNA (200 μM) (Sigma-Aldrich) by using a microplate reader (monitored at 405 nm).

### Statistical analysis

The data are expressed as the mean ± s.d. Differences were analyzed using dependent or independent t-tests. Values of *P* < 0.05 were considered significant.

## Electronic supplementary material

SUPPLEMENTARY INFORMATION
