# Supplementary material for: Anti-leukemia activity of a Hsp70 inhibitor and its hybrid molecules
Source: Sci Rep. 2017 Jun 14;7:3537. doi: 10.1038/s41598-017-03814-6 (PMC5471252; doi:10.1038/s41598-017-03814-6)
Supplement: Supplementary file 1 — SUPPLEMENTARY INFORMATION [file 41598_2017_3814_MOESM1_ESM.doc]

**SUPPLEMENTARY INFORMATION**

**Anti-leukemia activity of a Hsp70 inhibitor and its hybrid molecules**

Seong-Hyun Park^1^, Won-Je Kim^2^, Hui Li^1^, Wonil Seo^1^, Sang-Hyun Park^1^, Hwan Kim^3^, Sang Chul Shin^4^, Erik R. P. Zuiderweg^5^, Eunice EunKyeong Kim^4^, Taebo Sim^3,6^, Nak-Kyoon Kim^2^,* and Injae Shin^1^,*

^1^National Creative Research Initiative Center for Biofunctional Molecules, Department of Chemistry, Yonsei University, Seoul 03722, Korea. E-mail: injae@yonsei.ac.kr

^2^Advanced Analysis Center, Korea Institute of Science and Technology (KIST), Seoul 02792, Korea. E-mail: nkkim@kist.re.kr

^3^Chemical Kinomics Research Center, Korea Institute of Science and Technology (KIST), Seoul 02792, Korea

^4^Biomedical Research Institute, Korea Institute of Science and Technology (KIST), Seoul 02792, Korea

^5^Department of Biological Chemistry, The University of Michigan, Ann Arbor, MI 48109, USA

^6^KU-KIST Graduate School of Converging Science and Technology, Korea University, Seoul 02841, Korea

**Supplementary Materials and Methods**

**Synthesis of hybrid molecules consisting of Az and geldanamycin (Az-GD, Az-O_3_-GD and Az-amide-GD)**

**Synthesis of Az-GD.** To a stirred solution of geldanamycin (56 mg, 0.1 mmol) in anhydrous DMF (1 mL) was added apoptozole (76 mg, 0.1 mmol) in anhydrous DMF (1 mL). After 24 h, the mixture was diluted with EtOAc (25 mL), washed with water (5 mL x 3) and brine (5 mL), dried over anhydrous Na_2_SO_4_, and concentrated under reduced pressure. The residue was subjected to flash column chromatography (CH_2_Cl_2_:MeOH = 20:1) to give **Az-GD** in 88% yield as a purple solid: ^1^H NMR (DMSO-*d*_6_, 400 MHz) δ 8.22 (s, 2 H), 8.10 (s, 1 H), 7.73 (d, *J* = 8.4 Hz, 2 H), 7.43 (d, *J* = 8.8 Hz, 2 H), 7.29 (d, *J* = 8.8 Hz, 2 H), 7.05-6.90 (m, 5 H), 6.83 (d, *J* = 8.8 Hz, 2 H), 6.75 (s, 1 H), 6.59 (t, *J* = 11.6 Hz, 1 H), 5.79 (m, 1 H), 5.53 (d, *J* = 9.6 Hz, 1 H), 5.25 (s, 2 H), 4.98 (s, 1 H), 4.40 (d, *J* = 9.6 Hz, 1 H), 3.78 (s, 3 H), 3.71 (s, 3 H), 3.66-3.46 (m, 10 H), 3.42-3.37 (m, 2 H), 3.25-3.17 (m, 6 H), 2.54 (m, 1 H), 2.27 (m, 1 H), 1.98-1.77 (m, 4 H), 1.63 (s, 3 H), 1.57-1.41 (m, 2 H), 0.98-0.88 (m, 3 H), 0.80 (m, 3 H); ^13^C NMR (DMSO-*d*_6_, 100 MHz) δ 184.2, 178.8, 169.6, 165.6, 159.7, 158.1, 156.2, 145.3, 143.2, 140.1, 137.6, 133.4, 133.0, 132.2, 130.8, 130.6, 130.5, 130.1, 128.3, 127.7, 127.4, 126.7, 125.9, 125.5, 124.4, 121.8, 121.7, 114.6, 113.7, 80.8, 79.8, 79.0, 72.1, 69.6, 69.5, 68.9, 68.8, 56.4, 55.9, 55.2, 55.0, 47.6, 44.5, 32.5, 30.7, 22.5, 13.0, 12.2; HR Q-TOF MS calcd for C_67_H_75_F_6_N_6_O_13_ [M+H]^+^ 1285.5291, found 1285.5287.

**Synthesis of Az-O_3_-GD.** To a stirred solution of geldanamycin (56 mg, 0.1 mmol) in anhydrous DMF (1 mL) was added Az-O_3_-NH_2_ (83 mg, 0.1 mmol) in anhydrous DMF (1 mL). After 24 h, the mixture was diluted with EtOAc (25 mL), washed with water (5 mL x 3) and brine (5 mL), dried over anhydrous Na_2_SO_4_, and concentrated under reduced pressure. The residue was subjected to flash column chromatography (CH_2_Cl_2_:MeOH = 20:1) to give **Az-O_3_-GD** in 82% yield as a purple solid: ^1^H NMR (CD_3_OD, 400 MHz) δ 8.09 (s, 2 H), 7.92 (s, 1 H), 7.89 (s, 2 H), 7.62 (d, *J* = 8.4 Hz, 2 H), 7.34 (d, *J* = 8.8 Hz, 2 H), 7.32 (d, *J* = 8.8 Hz, 2 H), 7.09-6.81 (m, 5 H), 6.71 (d, *J* = 8.8 Hz, 2 H), 6.55 (m, 1 H), 5.84-5.72 (m, 1 H), 5.60-5.45 (m, 1 H), 5.18 (s, 2 H), 5.11-5.05 (m, 1 H), 4.42 (d, *J* = 9.6 Hz, 1 H), 3.71 (s, 3 H), 3.66 (s, 3 H), 3.60-3.44 (m, 13 H), 3.41-3.32 (m, 3 H), 3.29-3.21 (m, 6 H), 3.19 (m, 2 H), 1.95-1.72 (m, 8 H), 1.63 (br. s., 3 H), 1.59-1.43 (m, 2 H), 0.95-0.83 (m, 6 H); ^13^C NMR (CD_3_OD, 100 MHz) δ 185.8, 181.0, 169.3, 164.9, 162.0, 160.4, 159.2, 146.9, 145.8, 143.0, 142.0, 140.1, 138.2, 135.5, 135.3, 134.5, 134.3, 133.7, 133.4, 133.1, 132.7, 132.4, 130.3, 130.0, 129.6, 129.0, 127.7, 127.3, 127.3, 126.0, 123.7, 123.3, 123.2, 123.1, 115.8, 114.8, 110.4, 109.3, 83.1, 82.7, 82.1, 74.3, 73.8, 71.7, 71.6, 71.5, 71.4, 71.3, 70.6, 70.5, 70.4, 70.3, 62.3, 62.0, 57.9, 57.6, 57.0, 56.0, 56.0, 55.8, 55.0, 45.3, 39.0, 37.1, 35.9, 34.7, 33.7, 31.8, 30.7, 30.4, 22.9, 13.7, 13.4, 12.6; HR Q-TOF MS calcd for C_71_H_82_F_6_N_6_O_14_ [M+H]^+^ 1357.5866, found 1357.5890.

**Synthesis of 1.** To a stirred solution of geldanamycin (112 mg, 0.2 mmol) in anhydrous DMF (1 mL) was added **2** (83 mg, 0.4 mmol) in anhydrous DMF (1 mL). After 24 h, the mixture was diluted with EtOAc (25 mL), washed with water (5 mL x 3) and brine (5 mL), dried over anhydrous Na_2_SO_4_, and concentrated under reduced pressure. The residue was subjected to flash column chromatography (CH_2_Cl_2_:MeOH = 10:1 to 5:1) to give **1** in 78% yield as a purple solid: ^1^H NMR (CD_3_OD, 400 MHz) δ 7.16 (d, *J* = 11.6 Hz, 1 H), 7.08 (s, 1 H), 6.65 (t, *J* = 11.6 Hz, 1 H), 5.90 (m, 1 H), 5.62 (d, *J* = 9.6 Hz, 1 H), 5.24 (s., 1 H), 4.56 (d, *J* = 9.6 Hz, 1 H), 4.15 (s, 1 H), 3.81-3.60 (m, 10 H), 3.52-3.45 (m, 1 H), 3.37-3.30 (m, 6 H), 3.04 (s, 2 H), 2.74 (m, 1 H), 2.43-2.25 (m, 2 H), 2.12-1.97 (m, 4 H), 1.91-1.83 (m, 1 H), 1.76 (s, 3 H), 1.67-1.55 (m, 1H), 1.05-0.83 (m, 6 H); ^13^C NMR (CD_3_OD, 100 MHz) δ 210.3, 185.9, 181.2, 170.7, 159.2, 146.9, 142.9, 138.1, 135.5, 134.6, 132.7, 129.8, 127.3, 110.4, 109.33, 83.1, 82.1, 74.4, 71.3, 71.2, 70.8, 70.5, 61.7, 57.7, 57.0, 46.1, 34.6, 33.7, 30.9, 25.4, 22.8, 14.6, 13.8, 12.6; ESI-MS calcd for C_36_H_53_N_3_O_13_ [M+Na]^+^ 758.3, found 758.7.

**Synthesis of Az-amide-GD.** To a stirred solution of **1** (74 mg, 0.1 mmol) in DMF (1 mL) was added 1-ethyl-3,3-dimethylaminopropyl carbodiimide (EDC, 21 mg, 0.11 mmol) and DMAP (1 mg, 0.01 mmol) at 0 ^o^C. After 10 min, apoptozole (76 mg, 0.1 mmol) in DMF (1 mL) was added to the solution. After stirring for 12 h at room temperature, the mixture was diluted with EtOAc (25 mL), washed with water (5 mL x 3) and brine (5 mL), dried over anhydrous Na_2_SO_4_, and concentrated under reduced pressure. The residue was subjected to flash column chromatography (CH_2_Cl_2_:MeOH = 20:1) to give **Az-amide-GD** in 72% yield as a purple solid: ^1^H NMR (CD_3_OD, 400 MHz) δ 8.20 (s, 2 H), 8.03 (s, 1 H), 7.74 (d, *J* = 8.4 Hz, 2 H), 7.43 (d, *J* = 8.8 Hz, 2 H), 7.27 (d, *J* = 8.8 Hz, 2 H), 7.21-6.90 (m, 6 H), 6.82 (d, *J* = 8.8 Hz, 2 H), 6.61 (t, *J* = 11.6 Hz, 1 H), 5.88 (m, 1 H), 5.62 (d, *J* = 9.6 Hz, 1 H), 5.51 (br.s., 1 H), 5.32-5.20 (m, 3 H), 4.54 (d, *J* = 9.6 Hz, 1 H), 3.99-3.93 (m, 2 H), 3.82 (s, 3 H), 3.76 (s, 3 H), 3.74-3.38 (m, 21 H), 3.31 (s, 3 H), 3.29 (s, 3 H), 2.73 (m, 2 H), 2.33 (m, 1 H), 1.97 (s, 3 H), 1.89-1.54 (m, 5 H), 1.01-0.99 (m, 6 H); ^13^C NMR (CD_3_OD, 100 MHz) δ 185.8, 181.2, 172.9, 170.8, 169.5, 162.0, 160.5, 159.2, 142.9, 146.9, 145.9, 142.9, 142.2, 140.1, 138.2, 135.5, 135.2, 134.6, 134.3, 133.8, 133.5, 133.1, 132.7, 132.5, 133.1, 132.5, 130.4, 129.6, 129.1, 127.7, 127.3, 126.0, 123.7, 123.3, 123.2, 115.8, 114.9, 110.4, 109.3, 83.1, 82.1, 74.5, 72.1, 71.6, 71.5, 71.4, 70.7, 70.3, 57.7, 57.0, 56.0, 55.8, 55.0, 46.4, 41.1, 39.9, 35.9, 34.7, 33.7, 22.9, 14.5, 13.8, 12.6; HR Q-TOF MS calcd for C_75_H_90_F_6_N_7_O_17_ [M+H]^+^ 1474.6292, found 1474.6294.

**Synthesis of hybrid molecules consisting of** **Az and imatinib** (**Az-O_3_-Imatinib** and **Az-O_4_-Imatinib**)

**Synthesis of 5 and 6*.*** To stirred solutions of **3** or **4**^1^ (1.34 mmol) and TEA (0.565 mL, 4.02 mmol) in CH_2_Cl_2_ (3 mL) was added Boc-piperazine (0.374 g, 2.08 mmol). The mixtures were stirred at reflux overnight and then concentrated under reduced pressure. The residues were subjected to flash column chromatography (EtOAc:hexane = 1:1) to afford **5** or **6** as colorless oils in 67.3% yield. Compound **5**: ^1^H NMR (CDCl_3_, 400 MHz) δ 3.62-3.50 (m, 12 H), 3.37-3.27 (m, 6 H), 2.51 (t, *J* = 5.6 Hz, 2 H), 2.35 (t, *J* = 4.4 Hz, 4 H), 1.36 (s, 9 H); ^13^C NMR (CDCl_3_, 100 MHz) δ 154.7, 79.5, 70.6, 70.6, 70.6, 70.3, 70.0, 68.8, 57.8, 53.3, 50.6, 28.4; ESI-MS calcd for C_17_H_33_N_5_O_5_ [M+H]^+^ 388.2, found 388.2. Compound **6**: ^1^H NMR (CDCl_3_, 400 MHz) δ 3.70-3.56 (m, 16 H), 3.41-3.34 (m, 6 H), 2.58 (t, *J* = 5.6 Hz, 2 H), 2.42 (m, 4 H), 1.43 (s, 9 H); ^13^C NMR (CDCl_3_, 100 MHz) δ 154.8, 79.6, 70.7, 70.6, 70.4, 70.1, 68.8, 57.8, 53.4, 50.7, 28.5; ESI-MS calcd for C_19_H_37_N_5_O_6_ [M+Na]^+^ 454.2, found 454.3.

**Synthesis of 7 and 8.** To stirred solutions of **5** or **6** (0.516 mmol) in anhydrous THF (1 mL) under argon atmosphere was added triphenylphosphine (0.162 g, 0.619 mmol) at 0 ^o^C. The mixtures were slowly warmed to room temperature. After 20 min, water (1 mL) was added to each solution. After 1 h, volatile materials were removed under reduced pressure. The residues were dissolved in ether and then washed with water. The organic layers were dried over anhydrous Na_2_SO_4_ and concentrated under reduced pressure to afford **7** or **8** as colorless oils in 94% yield. Compound **7**: ^1^H NMR (CDCl_3_, 400 MHz) δ 3.67-3.60 (m, 10 H), 3.51 (t, *J* = 5.2 Hz, 2 H), 3.43 (t, *J* = 5.2 Hz, 4 H), 2.86 (t, *J* = 5.6 Hz, 2 H), 2.59 (t, *J* = 5.6 Hz, 2 H), 2.44 (t, *J* = 5.2 Hz, 4 H), 1.45 (s, 9 H); ^13^C NMR (CDCl_3_, 100 MHz) δ 154.9, 132.4, 132.3, 132.1, 128.8, 128.6, 79.8, 73.7, 70.8, 70.8, 70.6, 70.5, 69.1, 58.0, 53.6, 42.0, 28.6; ESI-MS calcd for C_17_H_35_N_3_O_5_ [M+H]^+^ 362.2, found 362.4. Compound **8**: ^1^H NMR (CDCl_3_, 400 MHz) δ 3.68-3.56 (m, 14 H), 3.51 (t, *J* = 5.2 Hz, 2 H), 3.45-3.40 (m, 4 H), 2.86 (t, *J* = 5.2 Hz, 2 H), 2.58 (t, *J* = 5.6 Hz, 2 H), 2.43 (m, 4 H), 1.44 (s, 9 H); ^13^C NMR (CDCl_3_, 100 MHz) δ 154.8, 79.7, 73.1, 70.6, 70.4, 68.7, 57.9, 53.4, 41.7, 28.5; ESI-MS calcd for C_19_H_39_N_3_O_6_ [M+H]^+^ 406.2, found 406.2.

**Synthesis of 9 and 10.** To stirred solutions of **A** (300 mg, 0.479 mmol) in anhydride DMF (1 mL) was added 1-ethyl-3,3-dimethylaminopropyl carbodiimide (EDC, 110 mg, 0.575 mmol) and DMAP (18 mg, 0.144 mmol) at 0 ^o^C. After 10 min, **7** and **8** (0.622 mmol) in anhydride DMF (1 mL) were independently added to each of the above solutions. After 12 h at room temperature, the mixtures were diluted with EtOAc (30 mL), washed with water (10 mL x 3) and brine (10 mL), dried over anhydrous Na_2_SO_4_, and concentrated under reduced pressure. The residues were subjected to flash column chromatography (CH_2_Cl_2_:MeOH = 10:1) to give **9** or **10** in 65% yield as colorless oils. Compound **9**: ^1^H NMR (CDCl_3_, 400 MHz) δ 8.08 (s, 2 H), 7.83 (s, 1 H), 7.75 (d, *J* = 8.4 Hz, 2 H), 7.52 (d, *J* = 8.8 Hz, 2 H), 7.18 (d, *J* = 8.8 Hz, 2 H), 6.96-6.88 (m, 4 H), 6.81 (d, *J* = 9.2 Hz, 2 H), 5.14 (s, 2 H), 3.83 (s, 3 H), 3.79 (s, 3 H), 3.70-3.53 (m, 14 H), 3.40 (t, *J* = 4.8 Hz, 4 H), 2.54 (t, *J* = 5.6 Hz, 2 H), 2.40 (t, *J* = 4.8 Hz, 4 H), 1.44 (s, 9 H); ^13^C NMR (CDCl_3_, 100 MHz) δ 166.8, 160.4, 158.8, 154.9, 144.4, 140.5, 139.2, 134.4, 133.2, 132.4, 132.4, 132.0, 130.6, 128.7, 128.2, 128.1, 126.8, 125.9, 124.6, 122.3, 121.9, 114.9, 113.9, 79.8, 70.7, 70.7, 70.5, 70,5, 70,0, 69.0, 58.0, 55.5, 55.4, 53.5, 48.4, 40.1, 28.6; ESI-MS calcd for C_50_H_57_F_6_N_5_O_8_ [M+H]^+^ 970.4, found 970.8. Compound **10**: ^1^H NMR (CDCl_3_, 400 MHz) δ 8.08 (s, 2 H), 7.82 (s, 1 H), 7.76 (d, *J* = 8.4 Hz, 2 H), 7.51 (d, *J* = 8.8 Hz, 2 H), 7.19 (d, *J* = 8.8 Hz, 2 H), 6.95 (d, *J* = 8.4 Hz, 2 H), 6.89 (d, *J* = 8.8 Hz, 2 H), 6.80 (d, *J* = 8.8 Hz, 2 H), 5.13 (s, 2 H), 3.82 (s, 3 H), 3.77 (s, 3 H), 3.57-3.54 (m, 18 H), 3.40 (t, *J* = 4.8 Hz, 4 H), 2.53 (t, *J* = 5.6 Hz, 2 H), 2.39 (t, *J* = 4.8 Hz, 4 H), 1.45 (s, 9 H); ^13^C NMR (CDCl_3_, 100 MHz) δ 166.8, 160.3, 158.8, 154.8, 144.3, 140.3, 139.1, 134.3, 133.0, 132.6, 132.4, 132.3, 130.6, 128.7, 128.2, 128.1, 126.7, 125.8, 124.5, 122.3, 121.8, 114.8, 113.9, 80.0, 70.6, 70.6, 70.6, 70.6, 70.4, 70.3, 70.0, 68.3, 57.5, 55.4, 55.3, 53.1, 48.3, 40.0, 28.2; ESI-MS calcd for C_52_H_61_F_6_N_5_O_9_ [M+H]^+^ 1014.4, found 1014.7.

**Synthesis of Az-O_3_-Imatinib** **and** **Az-O_4_-Imatinib.** To stirred solutions of **9** or **10** (0.055 mmol) in CH_2_Cl_2_ (1 mL) was added TFA (1 mL). After 1 h, volatile materials were removed under reduced pressure to give **11** or **12**. The crude products were used in the next step without further purification.

To stirred solutions of **11** or **12** (0.034 mmol) and Cs_2_CO_3_ (0.034 g 0.10 mmol) in DMF (1 mL) under nitrogen atmosphere was added **B**^2^ (0.018 g, 0.041 mmol). After 12 h, the mixtures were diluted with EtOAc (30 mL), washed with water (10 mL x 3) and brine (10 mL), dried over anhydrous Na_2_SO_4_, and concentrated under reduced pressure. The residues were subjected to flash column chromatography (CH_2_Cl_2_:MeOH = 10:1) to give **Az-O_3_-Imatinib** or **Az-O_4_-Imatinib** in 52% yield as an orange solid. **Az-O_3_-Imatinib**: ^1^H NMR (acetone-*d*_6_, 400 MHz) δ 9.63 (s, 1 H), 9.10 (d, *J* = 7.6 Hz, 1 H), 8.95 (s, 1 H), 8.64 (d, *J* = 5.2 Hz, 1 H), 8.59 (s, 1 H), 8.40 (s, 2 H), 8.20 (s, 1 H), 8.06 (d, *J* = 8.0 Hz, 2 H), 7.82 (d, *J* = 8.4 Hz, 2 H), 7.68 (d, *J* = 7.6 Hz, 2 H), 7.62 (d, *J* = 5.2 Hz, 1 H), 7.50 (d, *J* = 8.8 Hz, 2 H), 7.47-7.44 (m, 1 H), 7.34 (d, *J* = 8.8 Hz, 2 H), 7.25 (d, *J* = 8.4 Hz, 1 H), 7.12 (d, *J* = 8.0 Hz, 2 H), 7.01 (d, *J* = 8.4 Hz, 2 H), 6.87 (d, *J* = 8.8 Hz, 2 H), 5.52 (s, 2 H), 4.42 (s, 2 H), 3.91-3.78 (m, 10 H), 3.68-3.44 (m, 20 H), 2.36 (s, 3 H); ^13^C NMR (acetone-*d*_6_, 100 MHz) δ 166.2, 166.1, 163.0, 162.3, 161.1, 159.3, 148.5, 146.0, 144.1, 141.2, 140.5, 138.8, 138.7, 138.5, 137.5, 136.0, 135.5, 133.9, 133.6, 133.3, 132.9, 132.2, 131.7, 131.2, 130.5, 129.9, 129.4, 129.1, 127.8, 127.6, 125.8, 125.2, 123.9, 123.0, 121.1, 118.1, 118.0, 117.4, 117.3, 117.2, 116.1, 115.2, 109.3, 71.4, 71.3, 71.3, 71.3, 70.6, 66.3, 60.8, 57.2, 56.2, 56.0, 51.2, 50.0, 49.7, 40.9, 18.2; HR MALDI-TOF MS calcd for C_69_H_68_F_6_N_10_O_7_ [M+Na]^+^ 1285.5069, found 1285.5059. **Az-O_4_-Imatinib**: ^1^H NMR (acetone-*d*_6_, 400 MHz) δ 9.61 (s, 1 H), 9.07 (d, *J* = 7.6 Hz, 1 H), 8.94 (s, 1 H), 8.64-8.578 (m, 2 H), 8.40 (s, 2 H), 8.18 (s, 1 H), 8.07 (d, *J* = 7.6 Hz, 2 H), 7.81 (d, *J* = 8.0 Hz, 2 H), 7.69 (m, 3 H), 7.51 (m, 3 H), 7.34 (d, *J* = 8.4 Hz, 2 H), 7.24 (d, *J* = 8.4 Hz, 1 H), 7.11 (d, *J* = 8.0 Hz, 2 H), 7.00 (d, *J* = 8.4 Hz, 2 H), 6.86 (d, *J* = 8.8 Hz, 2 H), 5.51 (s, 2 H), 4.39 (s, 2 H), 3.92-3.78 (m, 10 H), 3.59-3.52 (m, 24 H), 2.36 (s, 3 H); ^13^C NMR (acetone-*d*_6_, 100 MHz) δ 166.1, 162.2, 161.2, 161.0, 159.4, 148.8, 146.2, 144.1, 140.8, 140.6, 138.8, 138.7, 138.5, 137.4, 136.3, 135.9, 135.5, 133.8, 133.2, 132.9, 132.2, 132.1, 131.7, 131.1, 130.9, 129.8, 129.4, 129.1, 127.8, 127.5, 125.8, 125.0, 124.3, 123.1, 121.4, 118.0, 117.9, 117.3, 117.2, 116.0, 115.2, 109.3, 71.5, 71.4, 71.4, 71.3, 71.2, 71.2, 70.7, 66.3, 60.8, 57.1, 56.2, 56.0, 51.2, 49.9, 49.8, 40.9, 18.2; HR MALDI-TOF MS calcd for C_71_H_72_F_6_N_10_O_8_ [M+H]^+^ 1307.5512, found 1307.5508.

**Protein purification for NMR study.** *E. coli* BL21 (DE3) cells were transformed with pET28a-human Hsc70 ATPase domain (1-386 residues). The cells were grown until OD_600_ = 0.6 in a minimal (M9) media containing 1 g/L ^15^NH_4_Cl. The expression of the protein was induced with 0.5 mM isopropyl-β-thiogalactopyranoside (IPTG, Invitrogen) at 18 ^o^C overnight. Cells were harvested by centrifugation and re-suspended in buffer containing 20 mM HEPES (pH 7.4), 150 mM NaCl, and 100 μM PMSF. After cells were broken by sonication, the cell lysate was centrifuged at 18,000 rpm for 40 min at 4 ^o^C, and the cell debris was discarded. The supernatant was loaded onto a nickel-chelated Hi-Trap column (GE Healthcare) and eluted with a linear gradient of 25-500 mM imidazole in 20 mM HEPES (pH 7.2) and 150 mM NaCl. The collected fractions were further purified by using a Blue Sepharose HiTrap column (GE Healthcare) and gel filtration chromatography on HiLoad 26/60 Superdex-75 column (GE Healthcare) pre-equilibrated with 20 mM HEPES (pH 7.4) and 150 mM NaCl. For NMR experiments, the purified protein was concentrated into 20 mM Tris-HCl, 25 mM KCl, 5 mM MgCl_2_ and 5 mM K_3_PO_4_ in water containing 10% (v/v) D_2_O (pH 7.2) by using Amicon concentrators.

**Measurement of hydrodynamic size distribution.** The purified ATPase domain of Hsc70 (final concentration: 75 μM) was mixed with various concentration of Az (a phosphate salt form) in 20 mM Tris–HCl, 5 mM MgCl_2_, 25 mM KCl, 5 mM K_3_PO_4_ containing 1% DMSO (pH 7.4). Measurements of hydrodynamic size distribution of the mixture were performed by using Zetasizer (Malvern Instrument, UK) at 25 °C.

**ATPase assays.** ATPase assays were conducted in 20 mM Tris-HCl, 10 mM MgCl_2_ and 100 mM KCl buffer solution mixed with 2 μg of an ATPase domain of Hsp70. Each compound (10, 20 and 40 μM) was added to the protein solution in a 96-well plate. After the mixture was placed in the plate for 30 min at room temperature, 10 μM ATP was added to the mixture and the mixture was incubated for 3 h at 37 °C. To measure ATPase activity, 10 μl of a 1:100 mixture of PiColorLock Gold reagent (Innova Biosciences) and an accelerator was added to each well. After 5 min at room temperature, 4 μL of a stabilizer was added to the mixture and then incubated for additional 30 min before taking measurement. The absorbance at 620nm was measured by using an Infinite® 200 PRO multimode microplate reader. The same procedure was used to measure ATPase activity of Hsp90 except concentrations of protein (1 μg) and ATP (1 mM).

**Abl kinase assays.** Abl kinase assays were conducted by Eurofins Pharma Discovery Services (UK Limited). Briefly, each compound was pre-incubated with 0.25 μg/mL of human recombinant Abl (expressed in insect cells) in 20 mM MOPS containing 1 mM EDTA, 0.01% Brij-35, 5% glycerol, 0.1% β-mercaptoethanol and 1 mg/mL BSA for 15 min at 37 °C. To the enzyme mixture was added 0.2 mg/mL of poly(Glu:Tyr), 10 μM ATP and 0.25 μCi [γ^33^P]ATP. After incubation for 30 min, 3% H_3_PO_4_ was added to the mixture to stop enzyme reactions.  An aliquot was removed and counted to determine the amount of [^33^P]poly(Glu:Tyr) formed by using a Trilux scintillation counter (Wallac Microbeta 12-detector model, Perkin Elmer).

**DNA fragmentation assays.** HL-60 and K562 cells were incubated with 5 or 10 μM Az for 24 h. The cells were lysed in a buffer containing 10 mM Tris, 1 mM EDTA, and 0.2% Triton X-100 (pH 8.0). Samples were incubated in 100 μg/mL RNase A (30 min, 37 ^o^C) and 100 μg/mL proteinase K (10 min, 56 ^o^C). The DNA was precipitated by addition of 0.5 M NaCl-isopropyl alcohol and washed with 70% ethanol. Samples were loaded on a 1.5% agarose gel and subject to electrophoresis at 100 V for 0.5 h in TBE (Tris/Borate/EDTA) buffer (0.5X). The DNA ladder was stained with RedSafe™ nucleic acid staining solution (Intron, Korea) and analyzed by using a G:BOX Chemi Fluorescent & Chemiluminescent Imaging System (Syngene).

**Western blot analysis.** Proteins were separated by 6–15% SDS-PAGE. mouse HSC70 monoclonal (1:1000, Santa Cruz Biotechnology), mouse HSP70 monoclonal (1:1000, StressGen Biotechnology), mouse HSP90 monoclonal (1:1000, Santa Cruz Biotechnology), rabbit caspase-9 polyclonal (1:1000, Santa Cruz Biotechnology), rabbit caspase-3 (H-277) polyclonal (1:1000, Santa Cruz Bio Technology), rabbit cleaved Caspase-3 (Asp175) polyclonal (1:1000, Cell Signaling Technology), rabbit PARP polyclonal (1:1000, Cell Signaling Technology), rabbit AIF polyclonal (1:1000, Santa Cruz Bio Technology), mouse APAF-1 monoclonal (1:1000, Santa Cruz Biotechnology), mouse cytochrome c monoclonal (1:1000, Biovision), and mouse β-actin (1:1000, Santa Cruz Bio Technology) antibodies were used as primary antibodies. Horse peroxidase-conjugated goat anti-rabbit IgG (1:2000, Santa Cruz Bio Technology) and goat anti-mouse IgG (1:2000, Santa Cruz Bio Technology) were used as the secondary antibodies. The blots were developed by using a West-ZOL® plus Western Blot Detection System (Intron Biotechnology Inc., South Korea). Western blot signals were analyzed by using a G:BOX Chemi Fluorescent & Chemiluminescent Imaging System.

**Immunocytochemistry**. HL-60 and K562 cells were incubated with indicated concentration of Az for indicated time at 37 ^o^C. Treated cells were fixed with 4% formaldehyde in PBS buffer for 15 min. The cells were incubated with rabbit AIF polyclonal antibody (1:200, Santa Cruz Bio Technology) for 1 h at room temperature followed by incubation with Alexa-Fluor 488 conjugated rabbit IgG (1:200, Invitrogen, Molecular Probes) for 1 hour at room temperature and mounted with DAPI (Invitrogen-Molecular Probes). The cells were imaged by microscopy microscopy using a LSM 700 instrument. Cell images were analyzed using the ZEN 2011 software.

**Isolation of cytosol and mitochondrial fractions.** HL-60 and K562 cells were incubated with 5 or 10 μM Az for 24 h. Mitochondrial and cytosol fractions of cells were prepared by using a mitochondrial/cytosol fractionation kit (Biovision). Cells were harvested by centrifugation at 600 × *g* for 5 min and washed twice with cold PBS buffer. The cells were re-suspended in 250 μL extraction buffer containing the protease inhibitor mixture and dithiothreitol (Biovision). After incubation on ice for 30 min, the cells were homogenized on ice and centrifuged at 700 × *g* for 10 min at 4 °C, and the supernatant was collected. The collected supernatant was centrifuged again at 10,000 × *g* for 30 min at 4 °C. The resulting supernatant was harvested and used as cytosolic fractions. The pellets were re-suspended and used as mitochondrial fractions.

**Supplementary References**

1. Schrigten, D. *et al*. A new generation of radiofluorinated pyrimidine-2,4,6-triones as MMP-targeted radiotracers for positron emission tomography. *J Med Chem* **55**, 223-232 (2012)

2. Liu, Y. F., Wang, C. L., Bai, Y. J., Han, N., Jiao, J. P. & Qi, X. L. A facile total synthesis of imatinib base and its analogues. *Org Process Res Dev* **12**, 490-495 (2008)

**Supplementary Table S1.** IC_50_ values for Az and imatinib induced death of cells expressing wild-type and mutant Bcr-Abl (mean ± s.d., n = 3).

**Supplementary Figures and Figure Legends**

**Supplementary Figure S1.** Hydrodynamic size distribution of a mixture of various concentrations of apoptozole (a phosphate salt form) and an ATPase domain of Hsc70 (75 μM) in Tris buffer (pH 7.4) containing 1% DMSO was measured by using dynamic light scattering analysis. Protein aggregates were formed when 200 μM Az was mixed with 75 μM ATPase domain of Hsc70.

**Supplementary Figure S2**. Chemical shift perturbation. (a) Residues with large chemical shift changes (Δδ_NH_ > 0.04 ppm) upon ADP binding to an ATPase domain of Hsc70 were mapped in orange on the structure (PDB entry 2E8A). (b) Chemical shift changes observed in 2D ^15^N-^1^H TROSY HSQC spectra of an ATPase domain of Hsc70 upon addition of ADP (left) and Az (right). The ratios of protein to ligand are 1:0 (blue) and 1:2 (orange) for ADP binding, and 1:0 (blue), 1:0.5 (green), 1:1 (yellow), 1:1.25 (magenta) and 1:1.5 (red) for Az binding, respectively. (c) Plot of chemical shift changes between ligand-free Hsc70 and Az-bound (blue) or ADP-bound (orange) Hsc70. Subdomains IA, IB, IIA and IIB are indicated with green, magenta, violet and yellow on the residue numbers. Unassigned residues do not show any Δδ_NH_ values.

**Supplementary Figure S3.** Cytotoxicity of each compound against various leukemia cells. Indicated leukemia cells were treated with various concentrations of each compound for 48 h. Cell death was measured by using an MTT assay (mean ± s.d., n = 3).

**Supplementary Figure S4.** Cytotoxicity of Az and imatinib against Ba/F3 cells expressing (a) wild-type and (b) mutant Bcr-Abl (T315I). Cells were treated with various concentrations of each compound for 24 h. Cell death was measured by using an MTT assay (mean ± s.d., n = 3).

**Supplementary Figure S5.** Az induces apoptosis in Leukemia. Flow cytometry of HL-60 and K562 cells were treated with 10 μM of Az for 24 h and then stained with (a) a mixture of fluorescein-annexin V and PI (annexin V binding versus PI uptake) and (b) JC-1. Shown in (b) is a dot plot of red fluorescence (FL2, JC-1 aggregate) versus green fluorescence (FL1, JC-1 monomer).

**Supplementary Figure S6.** Az induces a large degree of cell shrinkage. (a) HL-60 and (b) K562 cells were treated with 10 μM Az for 24 h and the cell size was then determined by using flow cytometry (FSC; forward scatter, SSC; side scatter).

**Supplementary Figure S7.** Az promotes DNA fragmentation in leukemia cells. HL-60 and K562 cells were treated with Az for 24 h. The DNA fragments were then visualized by staining with RedSafe™ Nucleic Acid Staining Solution.

**Supplementary Figure S8.** Apoptotic processes. (a) During caspase-dependent apoptosis, cytochrome c is released from the mitochondria into the cytosol. The released cytochrome c binds to Apaf-1 to generate the apoptosome. The complex of cytochrome c and Apaf-1 activates caspase-9 through the proteolytic cleavage of procaspase-9. Subsequently, active caspase-9 induces activation of caspase-3 through the cleavage of procaspase-3. This event leads to degradation of DNA. Hsp70 suppresses the apoptosome formation by direct binding of a substrate binding domain of Hsp70 to Apaf-1. (b) During AIF-mediated caspase-independent apoptosis, apoptosis-inducing factor (AIF) is translocated from the cytosol to the nucleus. This event leads to degradation of DNA. Hsp70 directly bind AIF to block its translocation to the nucleus.

**Supplementary Figure S9.** Az blocks association of Hsp70 with Apaf-1. (a) HL-60 and K562 cells were treated with Az for 24 h, and the amount of Hsp70, Hsc70 and Apaf-1 was measured by western blot. Below graph shows quantitative analysis of each protein level (mean ± s.d., n = 3). (b) HL-60 and K562 cells were treated with Az for 24 h. Immunoprecipitation was performed with a Hsp70 antibody, and the amount of co-precipitated Apaf-1 was determined by western blot.

**Supplementary Figure S10.** Inhibitory effect of hybrid molecules on Hsp70, Hsp90 and Abl activities. (a) ATPase activity of Hsp70 was measured by using a Pi-Colorlock assay after incubation of an ATPase domain of Hsp70 with various concentrations of indicated molecules and 10 μM ATP (mean ± s.d., n =3). (b) ATPase activity of Hsp90 was measured by using a Pi-Colorlock assay after incubation of Hsp90 with various concentrations of indicated molecules and 1 mM ATP (mean ± s.d., n = 3). (c) Abl kinase activity was measured by using a radiometric kinase assay after incubation of Abl with a peptide substrate in the presence of various concentrations of indicated molecules and γ-^33^P-ATP (mean ± s.d., n =3).

**Supplementary Figure S11.** Cytotoxicity of hybrid compounds. Indicated leukemia cells were treated with various concentrations of each compound for 48 h. Cell death was measured by using an MTT assay (mean ± s.d., n =3).

**Supplementary Figure S12.** Az-O_3_-GD induces apoptosis in Leukemia. Flow cytometry of (a) HL-60 and (b) K562 cells treated with 10 μM Az, 10 μM GD or 4 μM Az-O_3_-GD for 24 h and then stained with a mixture of fluorescein-annexin V and PI (annexin V binding versus PI uptake) or stained with JC-1 (red fluorescence (FL2, JC-1 aggregate) versus green fluorescence (FL1, JC-1 monomer)). Cell size was also determined by using flow cytometry (FSC; forward scatter, SSC; side scatter). Untreated cells are used as a negative control.

**Supplementary Figure S13.** K562 cells were pre-incubated with 20 μM ZVAD-FMK for 3 h, and then treated with various concentrations of Az-O_3_-GD for 24 h. Effect of a caspase inhibitor on cell survival was determined by using an MTT assay (mean ± s.d., n = 3).

**Supplementary Figure S14.** HL-60 and K562 cells were treated with 2 μM Az-O_3_-GD for 24 h. (Top) Immunocytochemistry and (bottom) western blotting were performed by using an anti-AIF antibody. The nuclei were stained with DAPI (scale bar = 10 μm).

NMR spectra of Az-GD


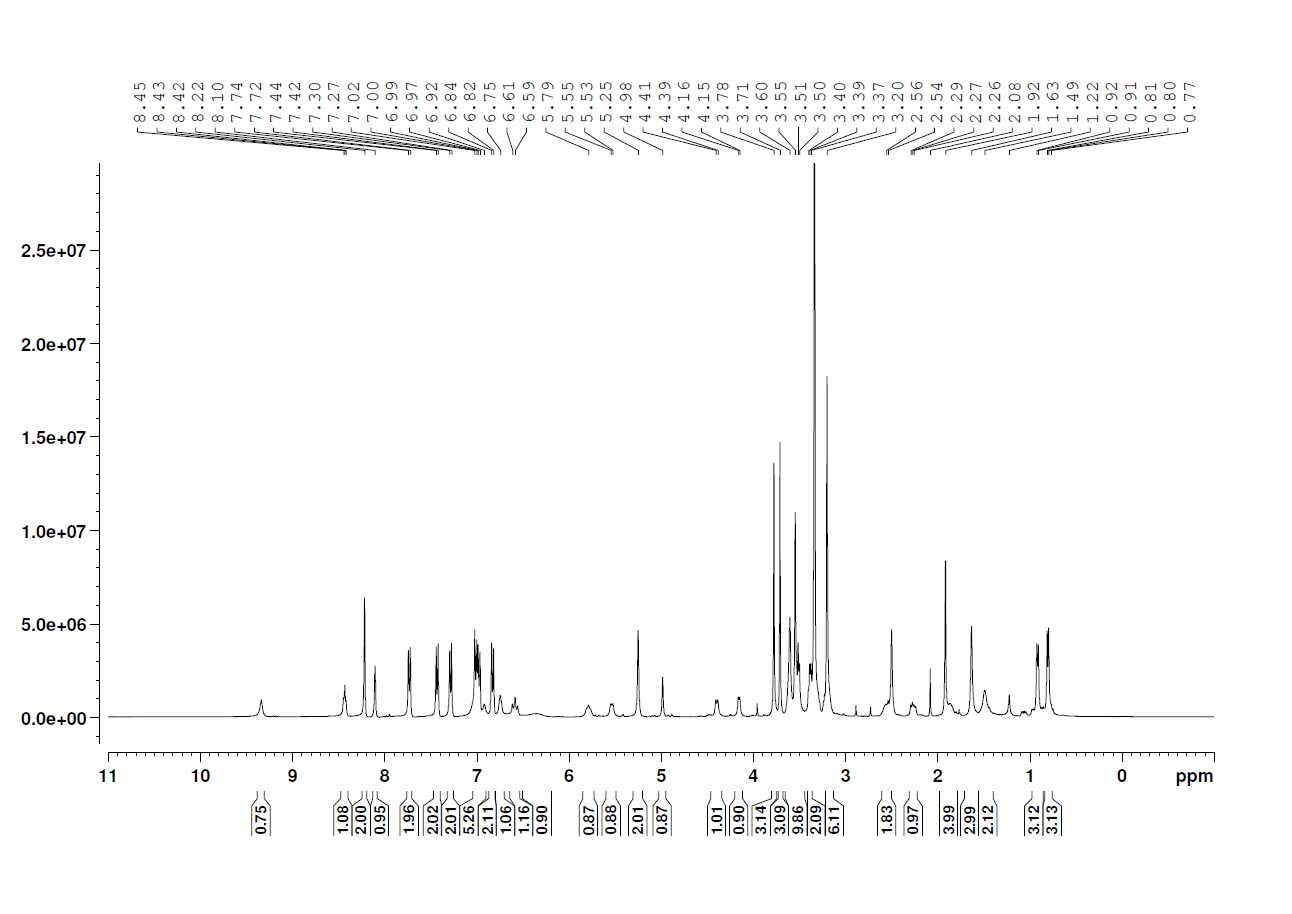


**Az-GD**

^1^H NMR (DMSO-*d_6_*, 400 MHz)

**
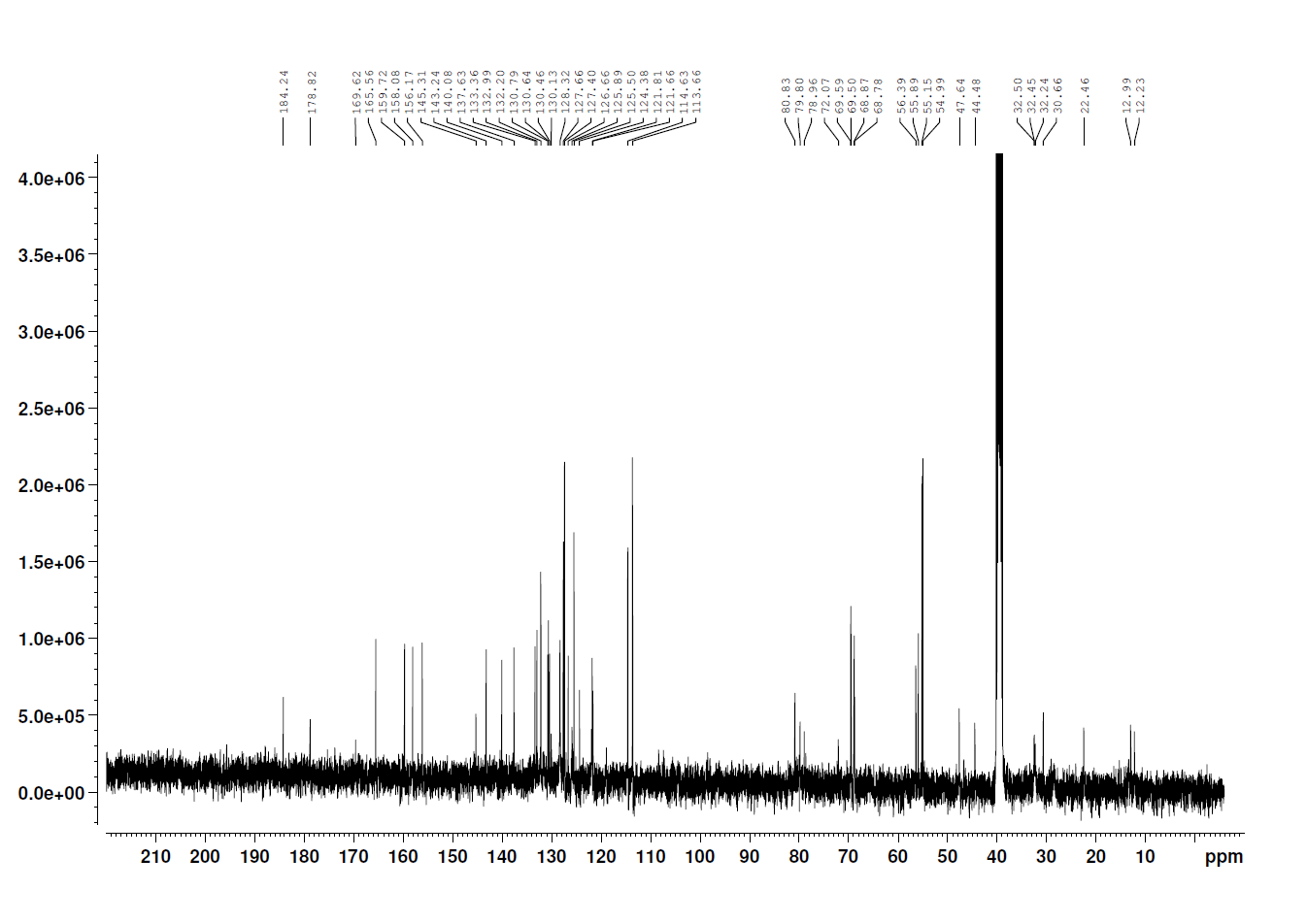
**

**Az-GD**

^13^C NMR (DMSO-*d_6_*, 100 MHz)

HPLC profile of Az-GD


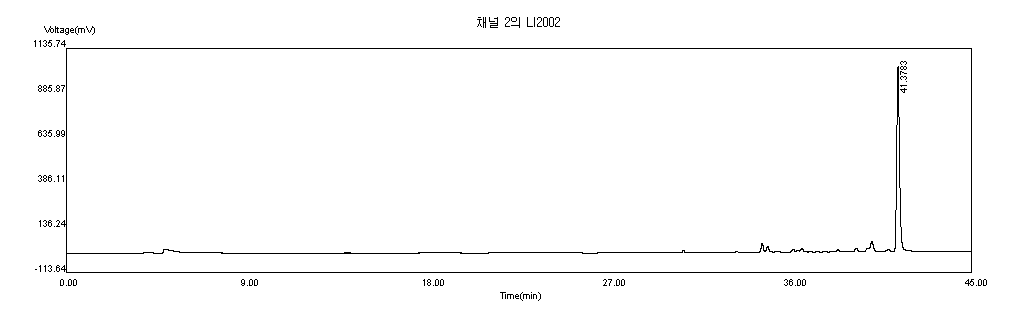


**9.00**

**18.00**

**27.00**

**36.00**

**45.00**

**Time( min)**

**Az-GD**

NMR spectra of Az-O_3_-GD


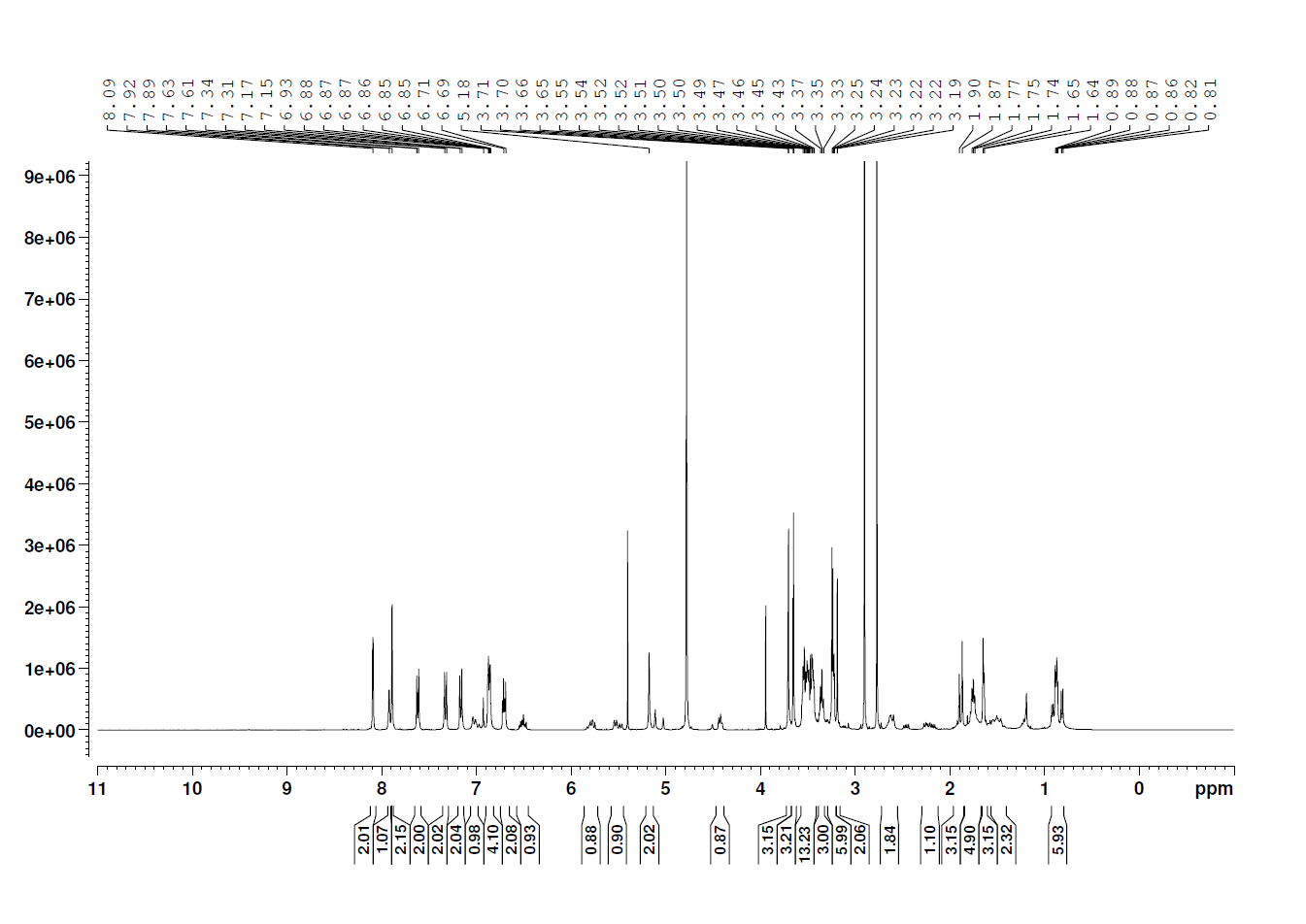


^1^H NMR (CD_3_OD, 400 MHz)

**Az-O_3_-GD**


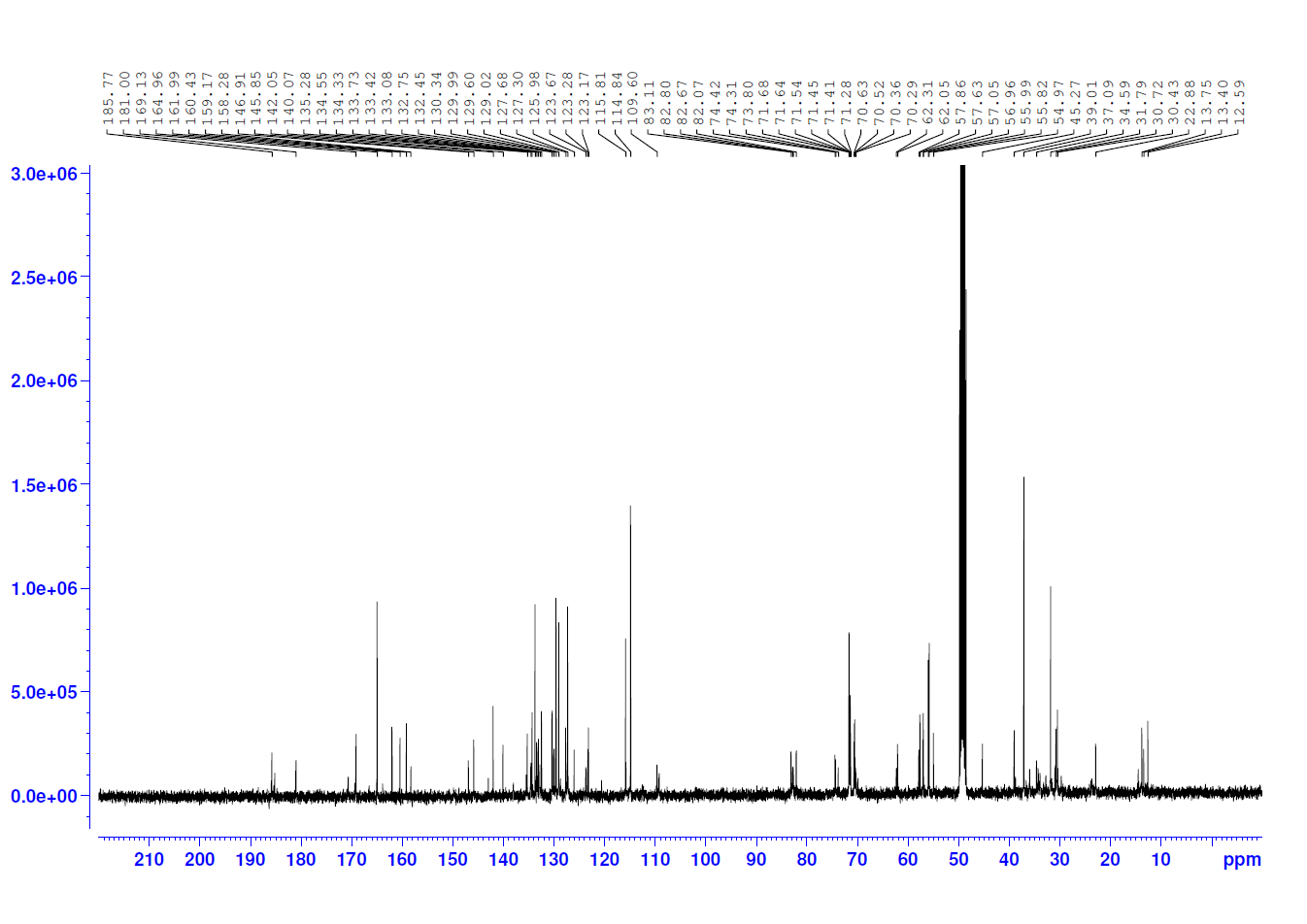


^13^C NMR (CD_3_OD, 100 MHz)

**Az-O_3_-GD**

HPLC profile of Az-O_3_-GD


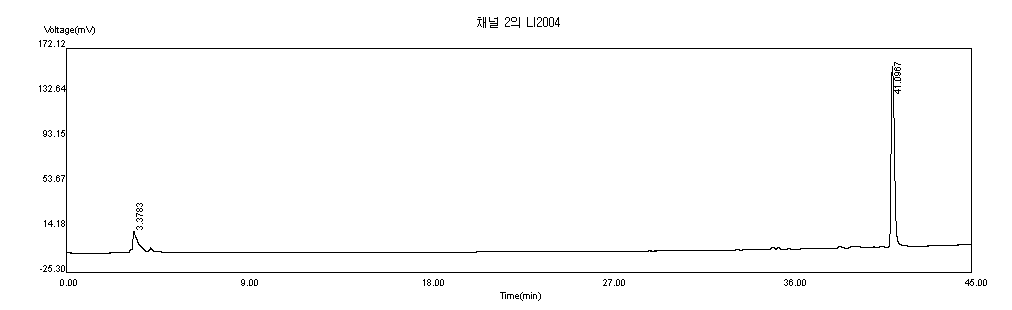


**9.00**

**18.00**

**27.00**

**36.00**

**45.00**

**Time (min)**

**Az-O_3_-GD**

NMR spectra of Az-amide-GD


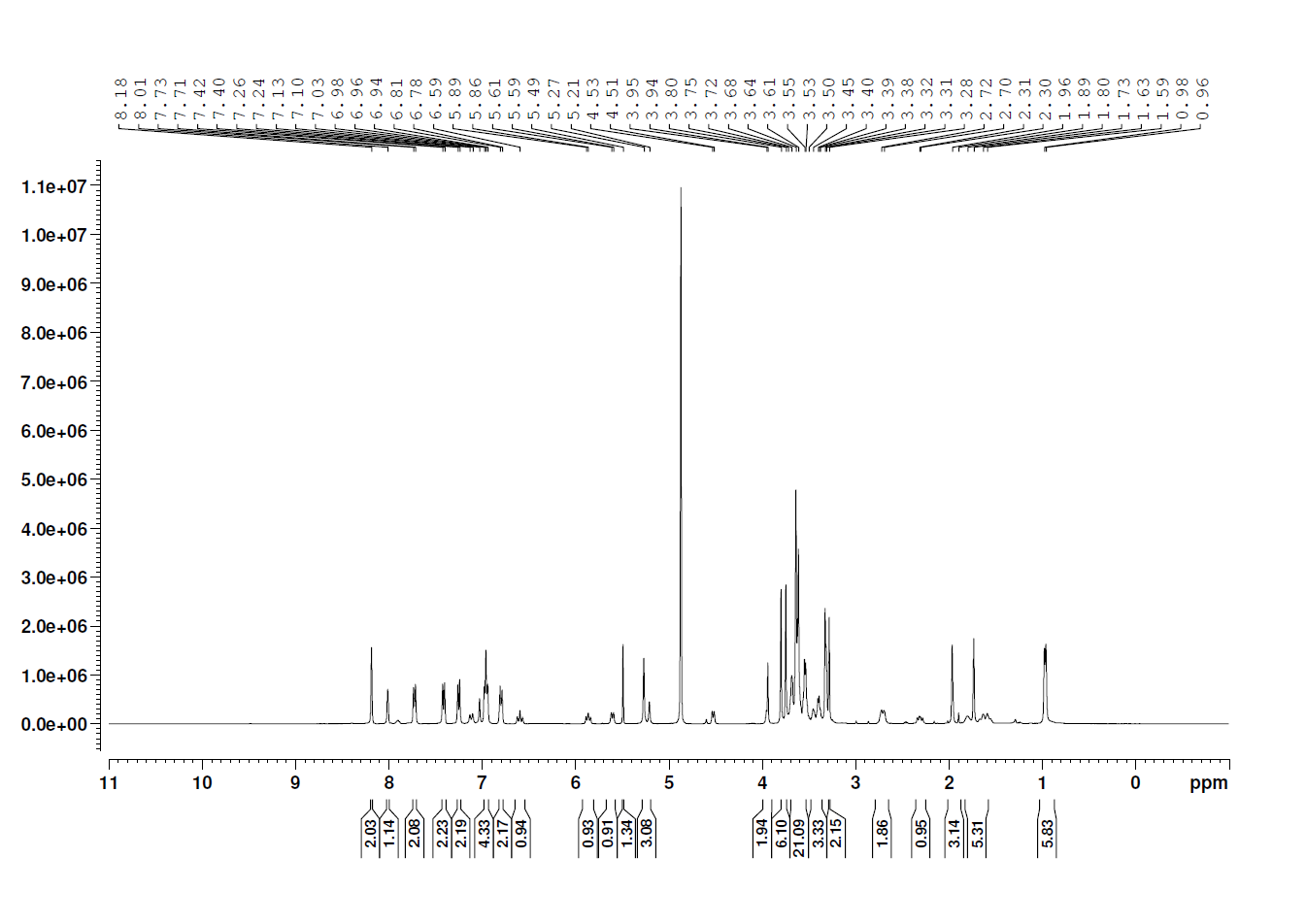


**Az-amide-GD**

^1^H NMR (CD_3_OD, 400 MHz)


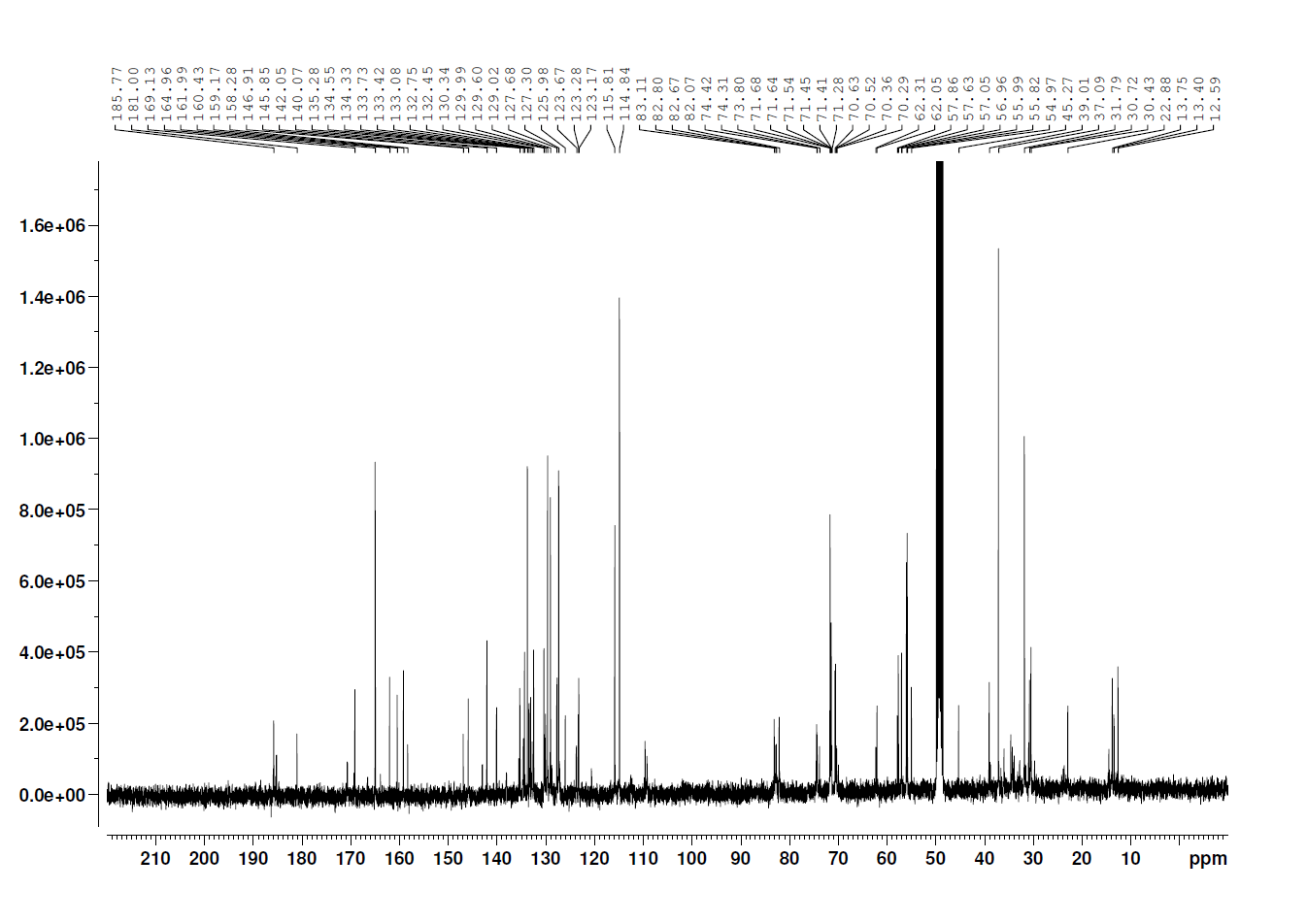


^13^C NMR (CD_3_OD, 100 MHz)

**Az-amide-GD**

HPLC profile of Az-amide-GD


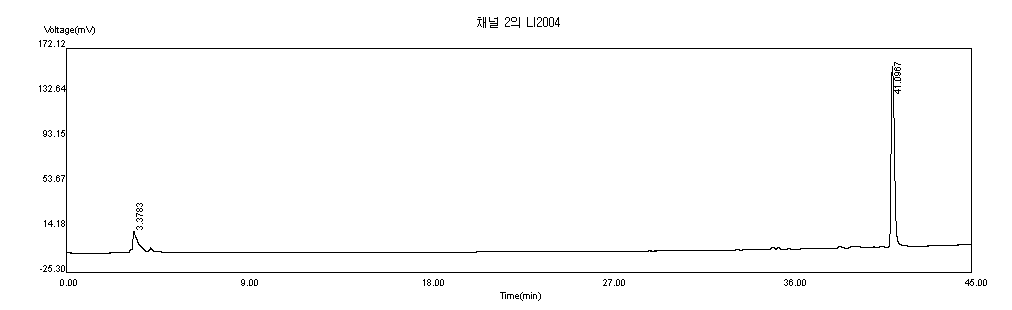


**9.00**

**18.00**

**27.00**

**36.00**

**45.00**

**Time (min)**

**Az-amide-GD**

NMR spectra of Az-O_3_-Imatinib


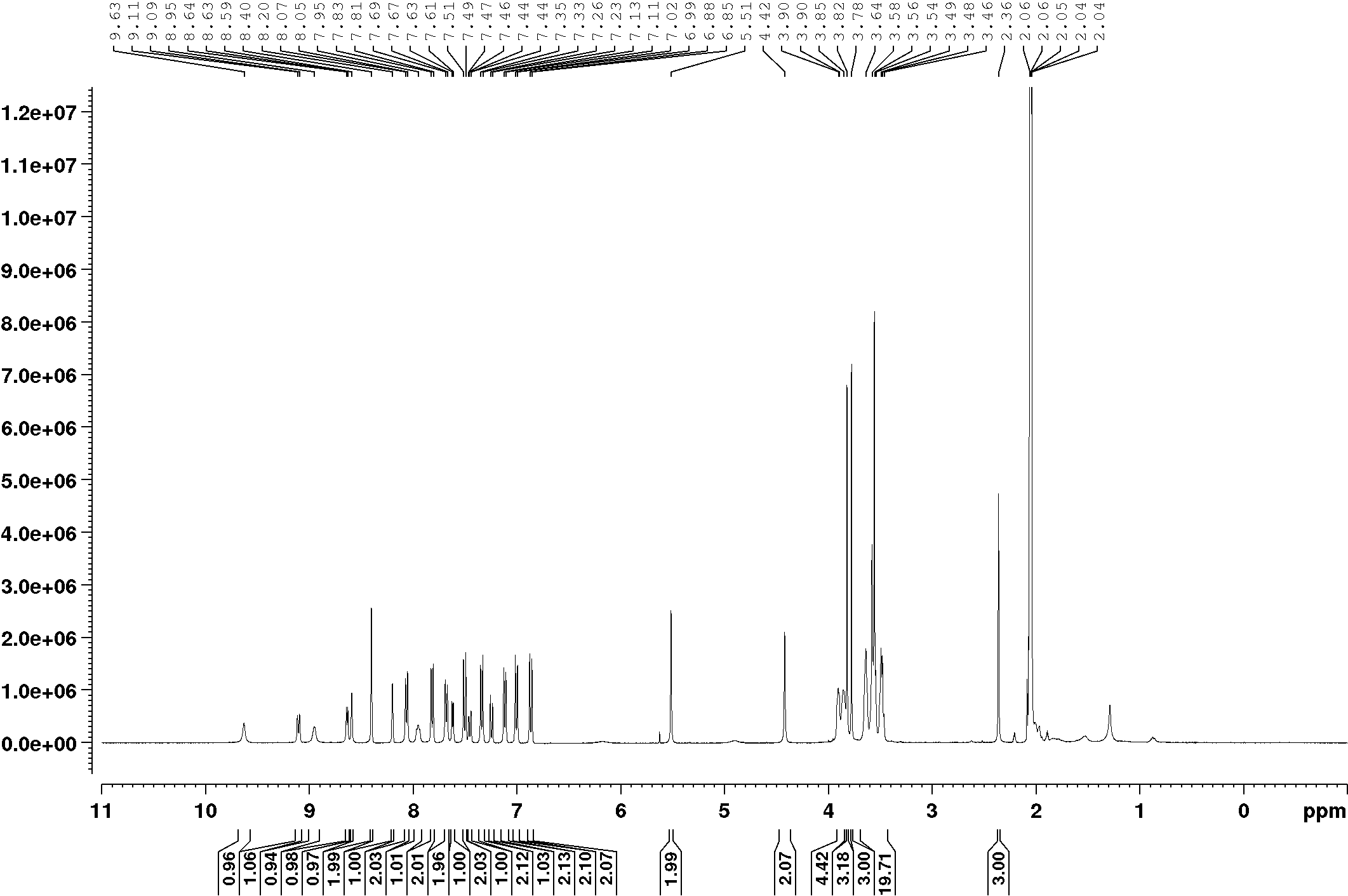


^1^H NMR (acetone-*d_6_*, 400 MHz)

**Az-O_3_-Imatinib**


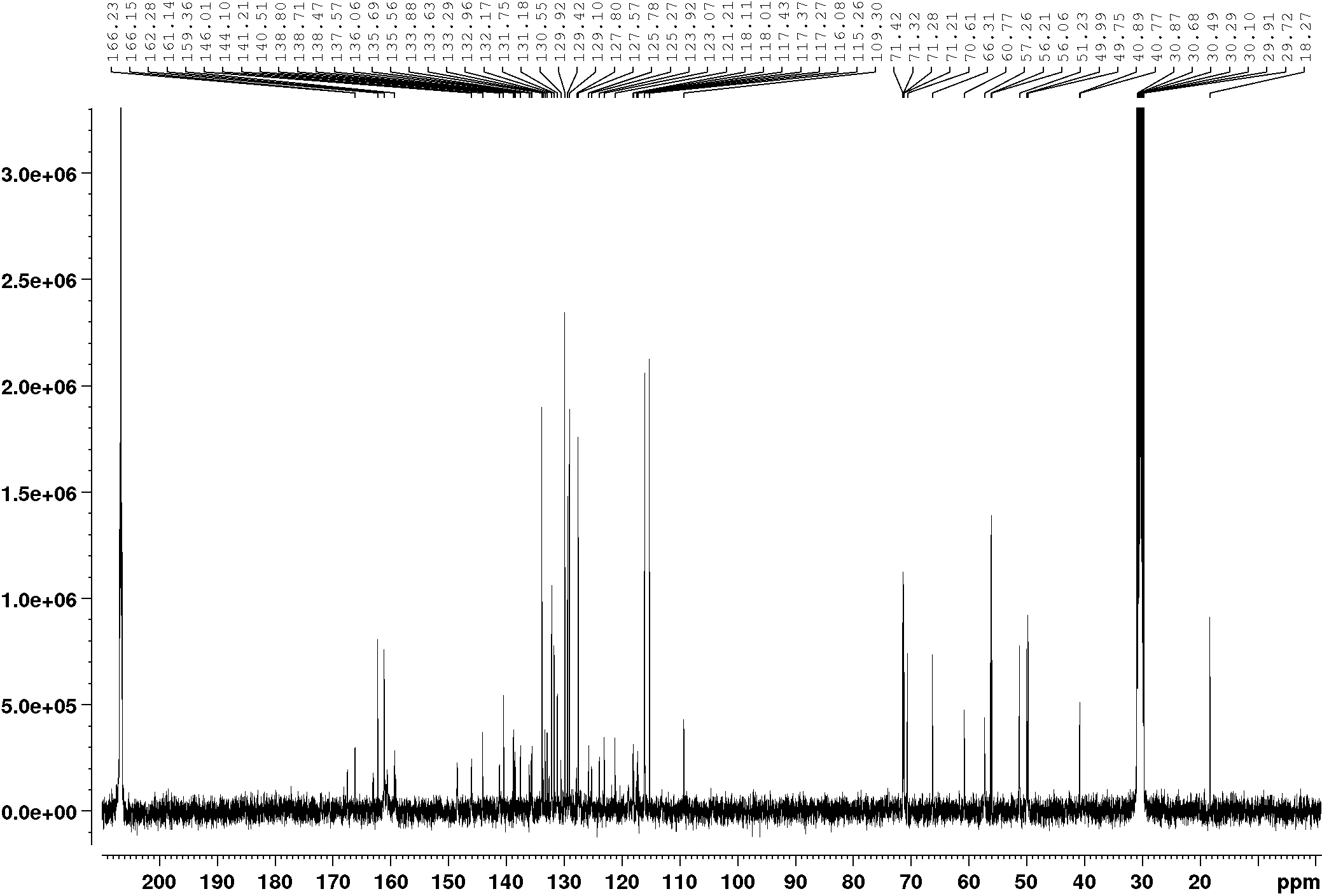


^13^C NMR (acetone-*d_6_*, 100 MHz)

**Az-O_3_-Imatinib**

HPLC profile of Az-O_3_-Imatinib


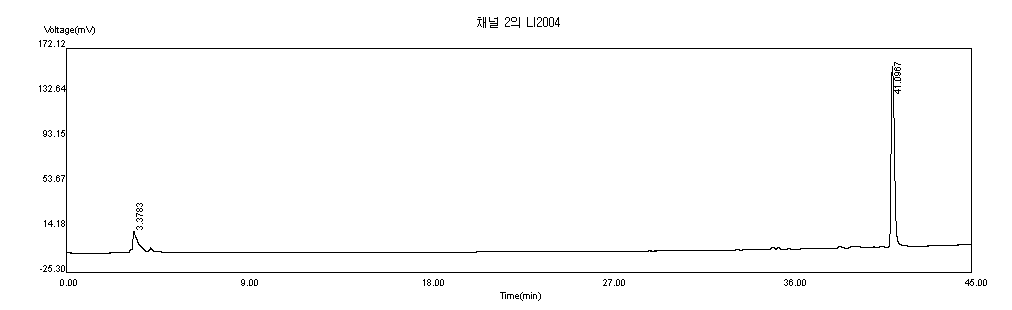


**9.00**

**18.00**

**27.00**

**36.00**

**45.00**

**Time (min)**

**Az-O_3_-Imatinib**

NMR spectra of Az-O_4_-Imatinib


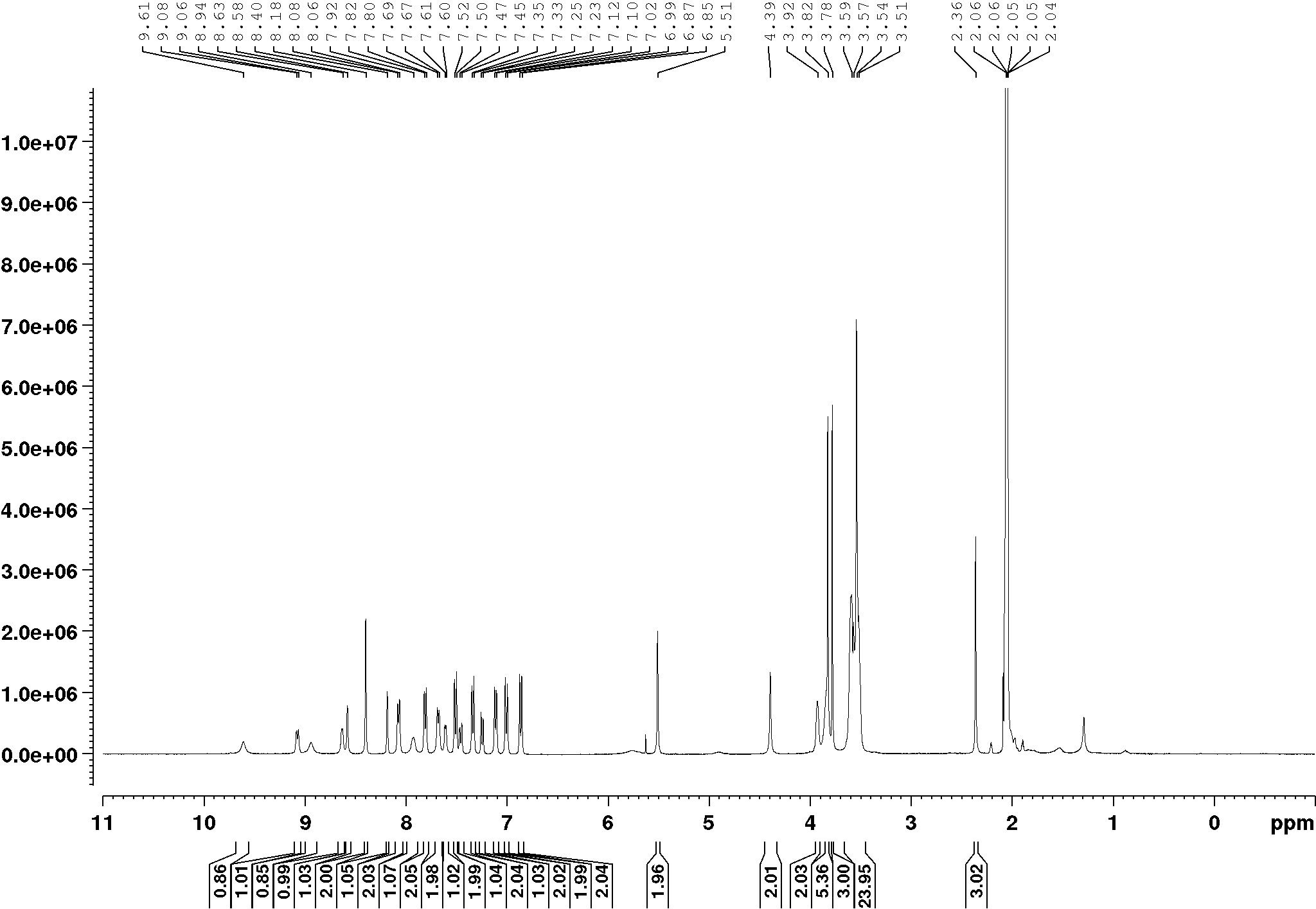


^1^H NMR (acetone-*d_6_*, 400 MHz)

**Az-O_4_-Imatinib**


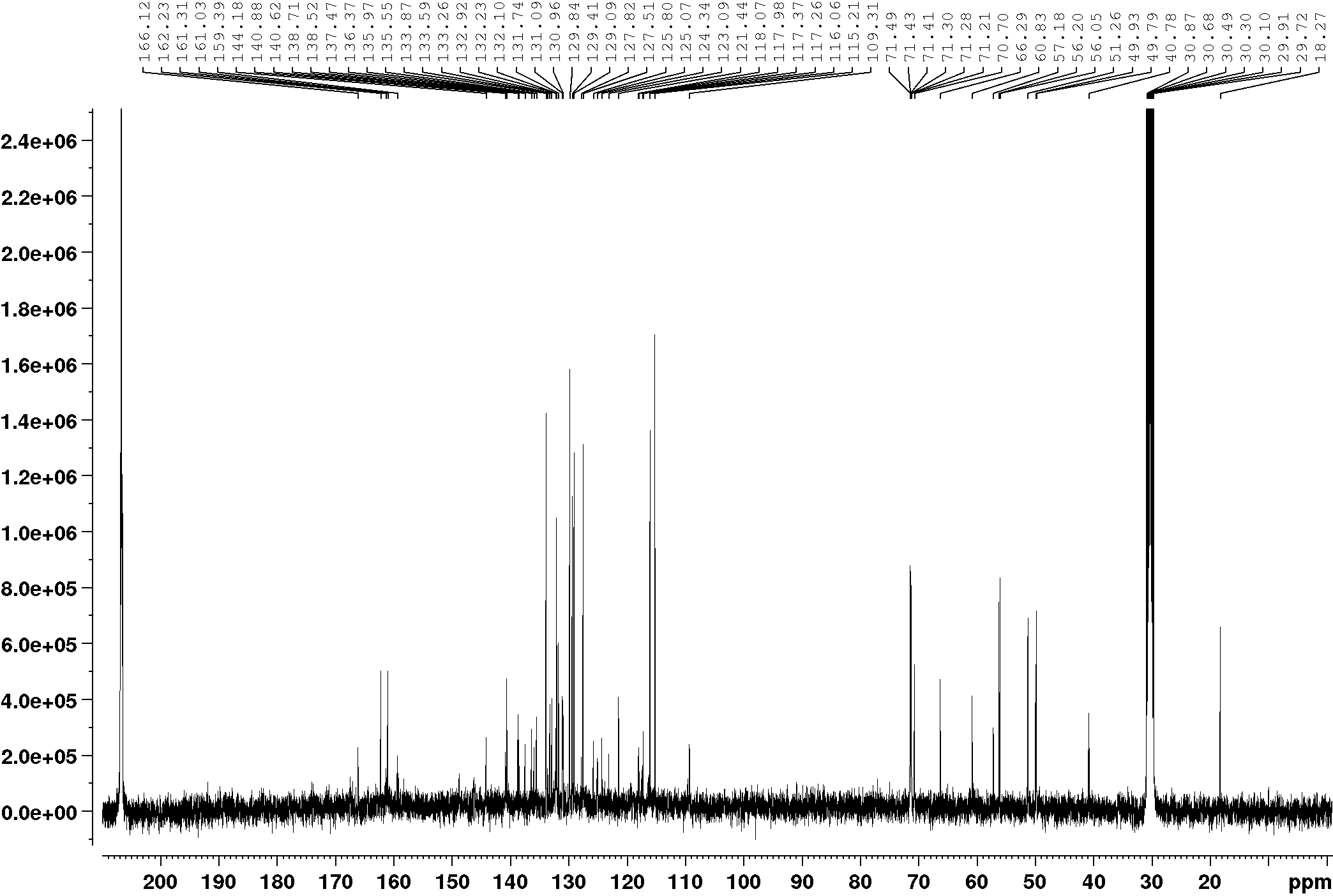


^13^C NMR (acetone-*d_6_*, 100 MHz)

**Az-O_4_-Imatinib**

HPLC profile of Az-O_4_-Imatinib

**9.00**

**18.00**

**27.00**

**36.00**

**45.00**

**Time (min)**


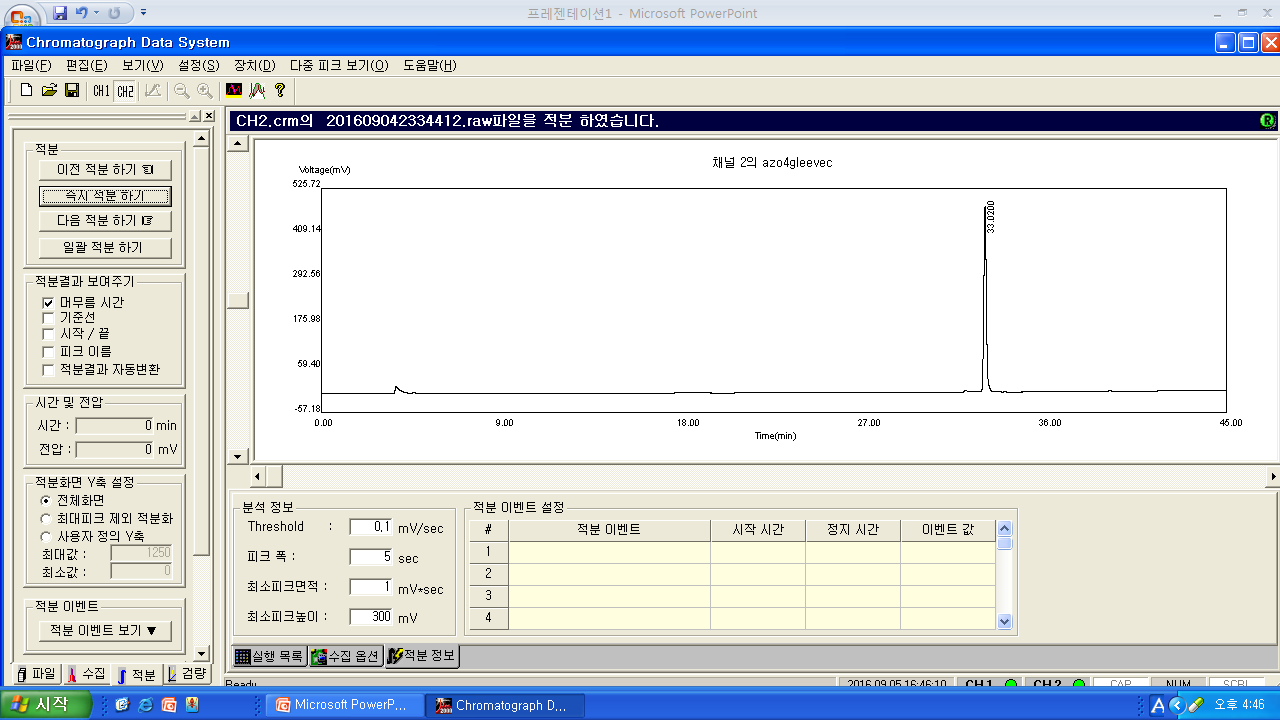


**Az-O_4_-Imatinib**
